# Single-cell phenomics reveals intra-species variation of phenotypic noise in yeast

**DOI:** 10.1186/1752-0509-7-54

**Published:** 2013-07-03

**Authors:** Gaël Yvert, Shinsuke Ohnuki, Satoru Nogami, Yasutaka Imanaga, Steffen Fehrmann, Joseph Schacherer, Yoshikazu Ohya

**Affiliations:** 1 Laboratoire de Biologie Moléculaire de la Cellule, Ecole Normale Supérieure de Lyon; CNRS, Université Lyon 1, 46 Allée d’Italie, Lyon F-69007, France; 2 Department of Integrated Biosciences; Graduate School of Frontier Sciences, University of Tokyo, Kashiwa, Chiba, Japan; 3 Department of Genetics, Genomics and Microbiology, University of Strasbourg; CNRS, Strasbourg, France

**Keywords:** Single-cell, *S. cerevisiae*, Yeast, Cell morphology, Stochasticity, Noise, Complex traits, Bet hedging

## Abstract

**Background:**

Most quantitative measures of phenotypic traits represent macroscopic contributions of large numbers of cells. Yet, cells of a tissue do not behave similarly, and molecular studies on several organisms have shown that regulations can be highly stochastic, sometimes generating diversified cellular phenotypes within tissues. Phenotypic noise, defined here as trait variability among isogenic cells of the same type and sharing a common environment, has therefore received a lot of attention. Given the potential fitness advantage provided by phenotypic noise in fluctuating environments, the possibility that it is directly subjected to evolutionary selection is being considered. For selection to act, phenotypic noise must differ between contemporary genotypes. Whether this is the case or not remains, however, unclear because phenotypic noise has very rarely been quantified in natural populations.

**Results:**

Using automated image analysis, we describe here the phenotypic diversity of *S. cerevisiae* morphology at single-cell resolution. We profiled hundreds of quantitative traits in more than 1,000 cells of 37 natural strains, which represent various geographical and ecological origins of the species. We observed abundant trait variation between strains, with no correlation with their ecological origin or population history. Phenotypic noise strongly depended on the strain background. Noise variation was largely trait-specific (specific strains showing elevated noise for subset of traits) but also global (a few strains displaying elevated noise for many unrelated traits).

**Conclusions:**

Our results demonstrate that phenotypic noise does differ quantitatively between natural populations. This supports the possibility that, if noise is adaptive, microevolution may tune it in the wild. This tuning may happen on specific traits or by varying the degree of global phenotypic buffering.

## Background

Modern biology is quantitative and scientists now pursue the exciting goal to link quantitative phenotypic variations to mechanistic molecular regulations. A frequent limitation in these investigations is the ability to accurately quantify the phenotype of interest. Tracking molecules and their abundance is sometimes not an issue, but defining and acquiring phenotypic traits precisely can be very demanding. In particular, most phenotypic measurements are made on macroscopic quantities reflecting the contribution of many cells. This is the case when describing tissue morphologies, growth rates of microorganisms, virulence of pathogens, yields of plants or the clinical outcome of a patient. However, rare cells, or heterogeneities among cells, may have important macroscopic consequences. Traits such as cancer, developmental defects, escape from drug treatment, or latency of infections can rely on one or few cells that did not follow the average behavior of a tissue. In these cases, quantifying biological traits at single-cell resolution is invaluable because it offers the possibility to link molecular variations to the microscopic sources of phenotypic variation. For example, an increased penetrance of a macroscopic trait may be associated to increased noise or to the presence of a stochastic switch, but finding this association requires to track the underlying mechanism in numerous individual cells [1,2]. Biologists will therefore gain enormous information from a statistical description of individual cells behaviors.

In particular, the potential fitness advantage that biological ‘noise’ may confer to organisms is frequently discussed. Intuitively, maintaining a diversified population of cells is costly in constant and unperturbed environments but can prove advantageous if the environment fluctuates, because a fraction of cells may then be readily adapted. Examples of a fitness advantage provided by stochastic switches were found for bacterial persistence under antibiotic exposures [3] and bacterial morphology or pigmentation under experimental evolution of dimorphism [4,5]. In addition, simulations have explored evolutionary scenarios that could explain the emergence of stochastic switching [6]. Importantly, evidence of positive selection for high noise was found for yeast genes coding for plasma-membrane transporters [7]. Yet, this discussion suffers from a central unanswered question: does phenotypic noise vary among different natural populations? From the effect of artificial mutations, some authors successfully classified gene products by their contribution to phenotypic buffering [8]. But what about natural alleles, which exist in the wild and through which evolution takes place? Do they also confer specific buffering capabilities? So far, only few examples suggest that they do. One is the fact that developmental asymmetry can be fixed using supervised crosses between natural fly stocks [9]. Another is the observation that noise in gene expression varies as a complex trait between natural genotypes of the yeast *S. cerevisiae*[10]. However, molecular noise can be buffered in various ways and does not necessarily generate phenotypic variation. Negative feedbacks can efficiently attenuate noise levels in gene circuits [11]. So can redundancy between molecular pathways: if two independent chains of reactions contribute to the phenotypic output, then molecular noise in only one chain may not affect the buffering provided by the other chain. It is therefore essential to directly track phenotypic noise levels in natural populations to determine whether they differ in the wild. If the answer is positive, then microevolution may take place to select for or against elevated noise. If negative, then selection for elevated noise first requires a step where genotypes generating higher noise or phenotypic switches appear in the population.

A preponderant model system for the study of cellular traits is the yeast *S. cerevisiae*[12]. Yet, obtaining robust quantitative estimates of phenotypic traits in this system can be very demanding if the trait is not directly coupled to a growth rate. In the case of cellular morphology and organization, this limitation was released some years ago by the development of a semi-automated protocol, which can profile hundreds of individual cells [13]. The method consists of a triple labelling of fixed cells to visualize their cell wall, DNA and actin by fluorescent microscopy. Images are automatically acquired and analyzed with a dedicated algorithm that extracts 501 quantitative parameters (distances, areas, intensities, angles and so on) that reflect various aspects of cellular morphology. This single-cell phenomics approach is extremely sensitive, as it was able to detect unsuspected trait variation among a collection of gene-deletion mutants [13].

Using this technique, we provide here a comprehensive quantification of hundreds of single-cell traits in numerous unrelated natural strains of *S. cerevisiae*. We found an abundant variation of cellular morphology and organization between strains. Morphological differences did not reflect the population history of the species. Importantly, the single-cell resolution of the dataset provides a direct observation that, indeed, phenotypic noise does vary between natural contemporary genetic backgrounds.

## Results

### Single-cell phenomics of unrelated wild strains

**Figure 1:**
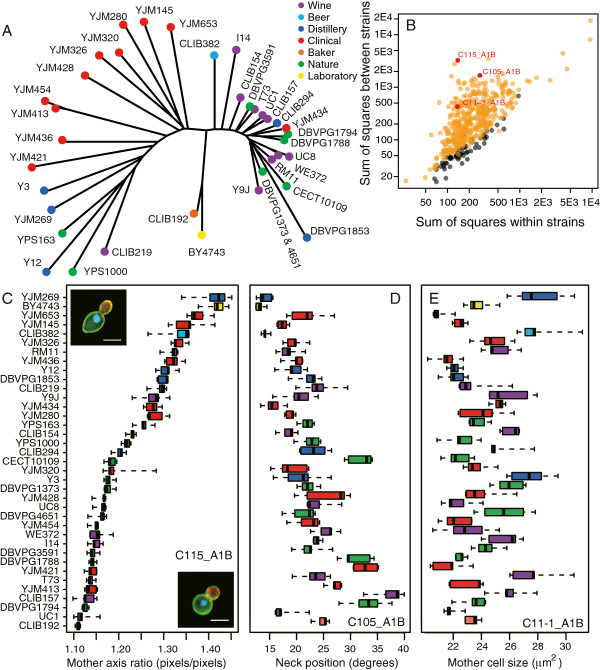
**Natural intra-species variability of *S. cerevisiae *cellular morphology. A)** The panel of strains used in this study is shown on a neighbour-joining tree reflecting genetic distances. Branch lengths are proportional to the fraction of 101,343 segregating sites that differentiate each pair of strains, as described in [14]. Colors reflect ecological annotations. **B)** Intra- versus inter-strain variability of morphological traits. Each dot represents one of 501 measured parameters. Orange and grey distinguish the traits that were called significant and non-significant by the Kruskal-Wallis test at FDR = 0.01, respectively. For the purpose of visual clarity, each parameter was transformed by f(x) = (x-μ_BY_) / σ_BY_, where μ_BY_ and σ_BY_ are the mean and standard deviations of the parameter across 34 replicates of the BY4743 strain. Note that significance inference was determined from ranks of raw values and was therefore not affected by this transformation. The sum of squares across replicates (x-axis) and across strains (y-axis) were then computed. The three parameters highlighted in red reflect distinct cellular properties : long over short axis ratio of the ellipse fitted to the mother cell (C115_A1B), angle of neck position (C105_A1B) and total mother cell size (C11-1_A1B). **C)** Boxplot representation of C115_A1B values for all strains. Box colors represent ecological origins as in **A)**. Insets show representative images of the two extreme strains YJM269 (top) and CLIB192 (bottom) where mother cells are elongated and round, respectively, with fluorescent labelling of actin (red), DNA (blue) and cell wall (green). Bar : 5 μm. **D-E)** Similar representation for the two other traits highlighted in **B)**. The strain order is the same in all three panels **C**, **D** and **E**.

To estimate the extent of natural variation for morphological traits within the *S. cerevisiae* species, we selected 37 wild strains from various geographical and ecological origins (Figure 1A and Additional file 1: Table S1). These strains belong to a larger panel which was previously used to explore the genetic diversity of the species [14]. We selected this subset of strains in such a way that 1) most ecological and geographical classes were represented, 2) genetic distances between selected strains reflected all *S. cerevisiae* subgroups, 3) all strains were *MATa/MATα* diploids originating from the selfing of a haploid spore and 4) liquid cell cultures of these strains contained predominantly unattached individual cells rather than flocculent aggregates or clumps of unseparated cells. This latter criterion was essential to enable semi-automated image analysis of individual cells. We cultured each strain as five biological replicates in standard laboratory conditions as previously described [15] (exponential growth, synthetic medium, 2% glucose, 30°C). Cells were then fixed with formaldehyde and their cell wall, nuclear DNA and actin were stained using specific fluorescent dies. Images of at least 200 cells per culture were acquired by fluorescent microscopy. These images were then analyzed using the *CalMorph* software [13] to quantify 501 parameters reflecting the size, shape, orientation, and intracellular organization of the cells. Altogether, more than 1,000 cells were acquired for each strain, allowing the statistical inference of intra-species variation.

### Cellular morphology varies greatly across the *S. cerevisiae* species

To directly test each of the 501 traits for intra-species variability, we performed a Kruskal-Wallis test on the null hypothesis of no strain effect. Results were compared with those obtained across 1,000 permutation tests where the 185 values of the trait were resampled. A total of 440 traits showed *K > 56* from the actual dataset, while the empirical False Discovery Rate (FDR) associated with this threshold was 0.01 (Figure 1B and Additional file 2: Table S2). Detecting so many differences (88%) across only 37 strains suggests that most of the morphological organization of *S. cerevisiae* cells is subjected to intra-species quantitative variation.

The most striking phenotypic variation was the elongation of cells. For example, mother cells of the baker strain CLIB192 were nearly round whereas those of YJM269, isolated from apple juice, were clearly elongated, with a long axis about 1.3 times longer than their short axis (Figure 1C). This axis ratio was highly variable across strains both before and during budding, and for both mothers and buds (Additional file 2: Table S2). Thus, its variation does not reflect different properties at specific stages of the cell cycle but inherent differences in cell shape across the various backgrounds.

Another trait that greatly varied across strains was the position of bud neck. Some strains such as YJM269, BY4743, CLIB382 or UC1 budded almost longitudinally along their long axis, whereas other strains such as YJM421, DBVPG1794 or CLIB157 initiated budding at angle positions reaching 30–40 degrees (Figure 1D). This suggests that molecular determinants of bud initiation, such as Bud9p, Bud8p [16] or the 12S polarisome [17] may have strain-specific localization patterns along the cell cortex.

The size of cells was also highly variable across strains (Figure 1E). This fully agrees with previous observations made on industrial strains [18].

Importantly, many traits that were highly variable were not correlated. This is particularly apparent on Figure 1C-E, where values of the three traits mentioned above ranked strains in three different orders. Thus, the natural variation of *S. cerevisiae* cellular morphology represents a set of multiple independent traits with different sources of variability. We then investigated further the properties of this variation using conventional tools of multidimensional analysis.

### Wild strains are continuously distributed in the phenome space

**Figure 2:**
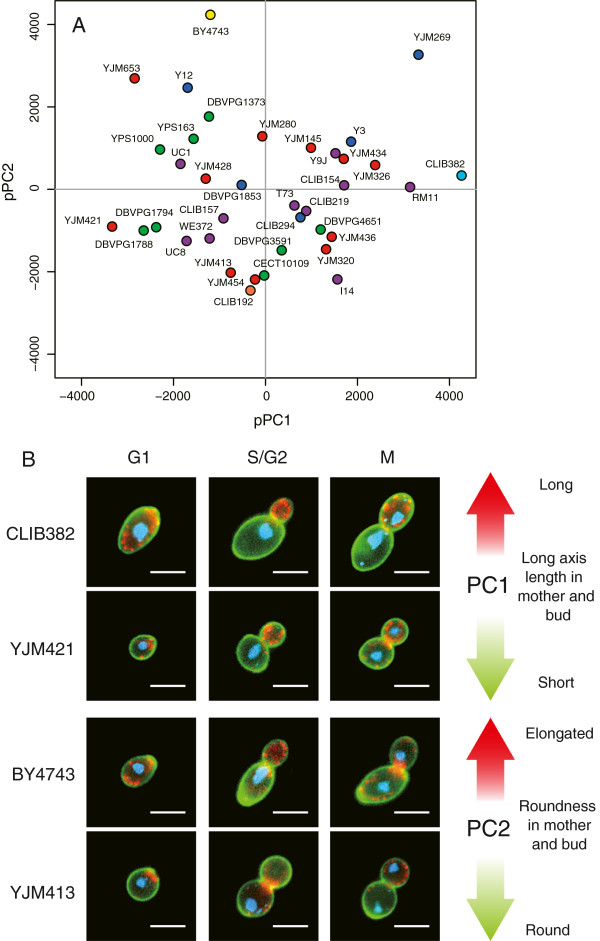
**Principal component analysis of *S. cerevisiae *morphological variation.** Raw trait values were transformed into their sum of ranks in each strain and then used for principal component analysis. **A)** Strains are represented by their coordinates along the first two principal components, using the same colors as in Figure 1A. **B**) Representative cells illustrating the traits contributing to the first two principal components. Bar: 5 μm.

Variation of multiple traits may take place in several ways. A first possibility is the existence of one or few strains showing peculiar morphologies compared to an overall profile globally conserved within the species. A second possibility is the co-existence of two or more distinct groups, each containing numerous strains. Finally, the morphological space may not be particularly structured, and strains may all differ continuously without presenting notable outsiders. To distinguish between these possibilities, we examined the overall landscape of phenotypic variations by performing principal component analysis (PCA). A permutation test determined that no principal component was expected to explain more than 5% of the variance by chance only. From the actual dataset, five phenotypic principal components (pPCs) were observed to exceed this threshold, and their cumulated contribution reached ~60% of the variance (Additional file 3: Figure S1). The first two components were contributed by traits reflecting cell elongation (Additional file 4: Table S3). After representing the position of strains along the first four components, several observations could be made (Figure 2A-B and Additional file 3: Figure S2). First, strains were almost evenly spaced with no particular subgroup that could explain any of the components. This reveals that *S. cerevisiae* has a continuum of morphological features rather than discrete classes of distinct morphologies. Secondly, strains from common ecological origin did not group together. This indicates that differences in the cellular traits measured do not simply reflect adaptation to the annotated environments. Less generally, adaptation could involve subtle changes of few traits. In this case, a dedicated test should be done to detect possible links between variation of one trait and the strain origin. We therefore tested, for every trait, the effect of ecological or geographical origin using a Kruskal-Wallis rank-sum test (see Methods). No significant association was found. This could be due to limited power in our small sample size (only 37 strains). It is also possible that some properties of the strains original microenvironments (pH, specific limiting nutrients or stress factors…) were shared between strains of similar morphological profiles. Detecting this possible adaptation would require exhaustive annotations of these environments at the time of collection. Finally, measuring morphological traits in a standardized laboratory condition may not interrogate the consequences of adaptation to specific environments. Acquiring morphological profiles from relevant ecological conditions would be more appropriate to reveal associations between traits and ecological origin.

**Figure 3:**
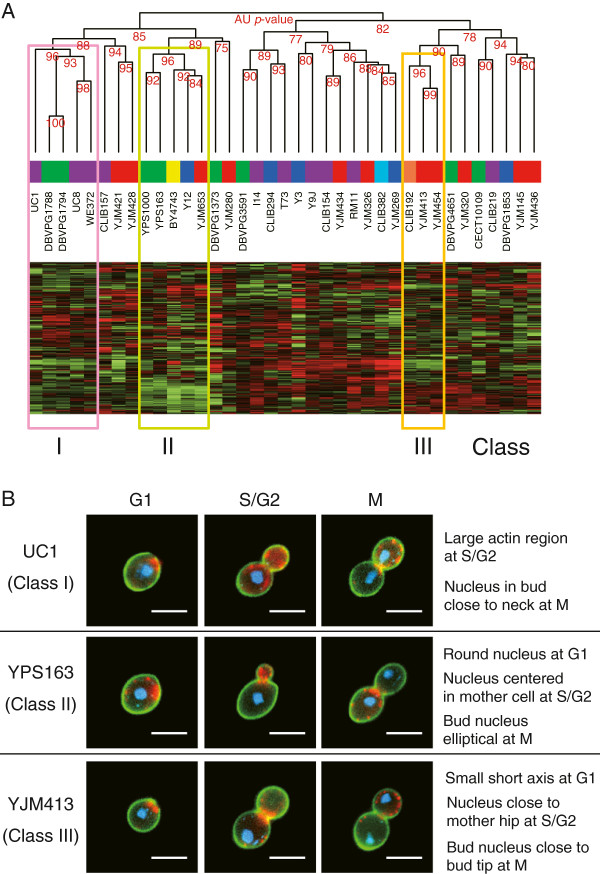
**Classification of strains based on morphological profiles. A)** Hierarchical clustering of strains. Numbers along the dendrogram indicate *p*-values computed by the multi-scale bootstrap technique [19] (see Methods). Colored squares over the strain names indicate their ecological origin, as in Figure 1A. The heatmap shows the variation of 501 traits from low (green) to high (red) values, with rows and columns representing traits and strains, respectively. Three significant classes of strains (I, II and III) are framed in color. **B)** Representative cells of each phenotypic class. The morphological features are described based on the parameters discriminating the classes (see Methods). Bar: 5 μm.

Although the overall landscape of trait variations was not structured, it remained possible that some subgroups of strains shared morphological similarities. To examine this possibility, we performed a classification based on hierarchical clustering and multiscale bootstrap resampling to infer statistical significance of the resulting dendrogram [19,20]. For each cluster, its derived approximately unbiased probability value (AU *p*-value) estimates the probability that the cluster would be observed if unlimited observations were available (i.e. infinite number of strains). The procedure defined three classes (I, II and III) of strains that were significantly grouped at AU *p*-value > 0.95 (Figure 3A and Additional file 3: Figure S3 and S4). Interestingly, each of the three classes contained strains from various ecological origins, indicating that the fine-scale structure detected could not simply be explained by shared environmental histories. To determine the phenotypic characteristics of these three classes, we performed a linear discriminant analysis (LDA, see Methods). This extracted 39, 9 and 19 parameters that significantly contributed to classes I, II and III, respectively (Additional file 5: Table S4). The main features of Class I were a large region of actin at S/G2 and a bud nucleus located close to the neck. Class II specificity was to display nuclei that were round and centered in mother cells but elliptical in buds. Class III contrasted by small cells at G1 and nuclei that were distant from the neck in both mother cells and buds (Figure 3B).

However, most strains (24 out of 37) remained unclassified, which is consistent with the continuous distribution of strains along the major principal components described above. Observing multiple singletons can sometimes result from high measurement errors. However, the high number of traits for which a significant strain effect could be detected indicates that our measures have small residual variance (Figure 1B). Thus, these numerous singletons more likely reflect that intra-species variation of *S. cerevisiae* cellular morphology is poorly structured.

### Relationship between phenotypic and genetic distances

**Figure 4:**
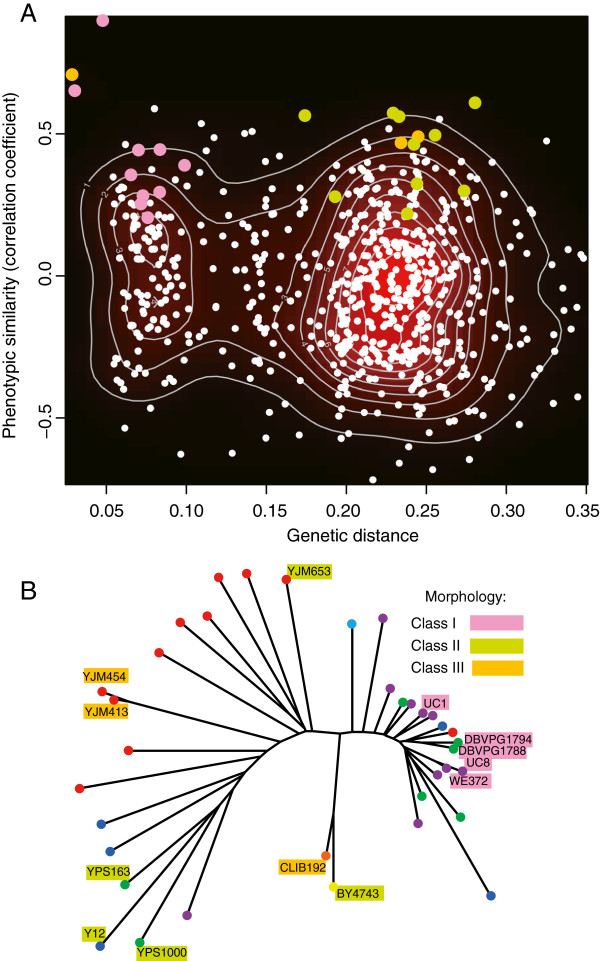
**Phenotypic versus genotypic distances. A)** Dot plot of pairwise distances between strains. Dots represent all 666 possible pairs of distinct strains, in color if the pair of strains corresponds to co-occurrence in phenotypic class I (pink), II (green) or III (yellow), and in white otherwise. Density of dots is highlighted by black to red colors and white density curves. *x*-axis: genetic distance computed as in Figure 1A. *y*-axis: phenotypic similarity. Every strain was associated to its coordinates along the first 28 principal components. Similarity between two strains was estimated by the Pearson correlation coefficient between the two vectors of coordinates associated to the strains. The two types of distances are not correlated (Spearman correlation between x and y values = −0.08). **B)** Position of strains from phenotypic classes I, II and III on the tree of genetic distances (same tree as in Figure 1A).

In order to study the relationship between genetic and phenotypic distances, we considered all 666 pairwise combinations of strains. Figure 4A represents their phenotypic similarity (defined as the Pearson correlation coefficient of the two strains across 28 pPC scores covering 97% of total variance in PCA on all 501 traits, see Methods) as a function of their genetic distance (defined as the number of polymorphic sites differentiating two strains, as previously described [14]). Except for three pairs of strains that were very close both genetically and phenotypically, there was absolutely no correlation between the two types of divergence (Spearman *ρ =* −0.08). Nevertheless, this absence of correlation could be due to the fact that our population/sample is a combination of strains coming from clean and mosaic lineages. By contrast to non-mosaic strains, mosaic isolates that are genetically distant might share common parts of the genome leading to a phenotypic similarity. We therefore examined correlation across 16 strains that were previously described to represent a clean lineage (see Methods). On this subset, genetic and phenotypic distances remained uncorrelated (Spearman *ρ = −*0.05), suggesting that our mixed population is not the major reason for not detecting any correlation. We conclude that, globally, morphological resemblance did not reflect genetic relatedness.

It still remained possible that subsets of traits co-varied with parts of the genetic structure of the population. To address this possibility, we extracted the principal components of the genotypic variance of the population (Additional file 3: Figure S5). The first component, gPC1, caught more than 25% of the variance and discriminated a cluster of European wine strains previously described [14]. The second component explained 7% of the variance and discriminated a pair of related clinical strains from the rest of the population. gPC3 and gPC4 explained about 5% of the variance each, and all successive ones had minor contributions. We then tested if these genotypic components of the population were correlated with any of the phenotypic principal components. We computed Spearman’s rank correlation coefficients among all combinations between the 37 gPCs and the 37 pPCs. None of these coefficients exceeded the correlations obtained when using pPCs from a randomized dataset. This implies that morphological traits and genotypic variations of this *S. cerevisiae* sample follow different structures.

When representing strains from classes I, II and III on the tree of genetic distances, we observed that class I strains were genetically close (Figure 4B). All five strains of class I belonged to a group of strains genetically related and generally associated with wine making [14]. The common features of these strains were to have large actin regions and a specific position of the nucleus (Additional file 5: Table S4). This suggests that phenotypic and genetic distances can be correlated locally. However, this was not the case for classes II and III. Class II contained strains YPS1000, BY and YJM653 that were all at different edges of the genetic tree, and class III contained clinical strain YJM454 and baker strain CLIB192 that were at extreme genetic distances from each other.

### Natural strains vary in their degree of cell-to-cell trait variation

**Figure 5:**
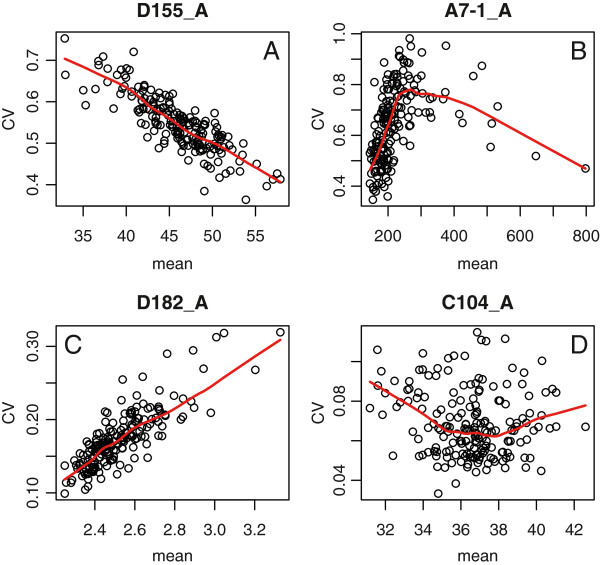
**Complex noise-vs-mean dependency of single-cell phenotypes.** Each plot represents 185 samples (5 per strain) of at least 200 cells each. *x*-axis: ‘mean’ value of the trait of interest in the sample. *y*-axis: coefficient of variation of the trait values within the sample. Four traits (indicated in headers) are shown as examples to indicate that the noise vs. mean dependency can be linear **(A** and **C)** or not **(B** and **D)**, and negative **(A)** or positive **(C)**. Red line: Lowess regression model used to condition noise on mean values. D155_A: Angle between [from cell center to brightest point in nucleus] and [end of major axis of the cell] at G1. A7-1_A: Size of actin region at G1. D182_A: Nuclear axis ratio at G1. C104_A: Short axis length in whole cell at G1.

**Figure 6:**
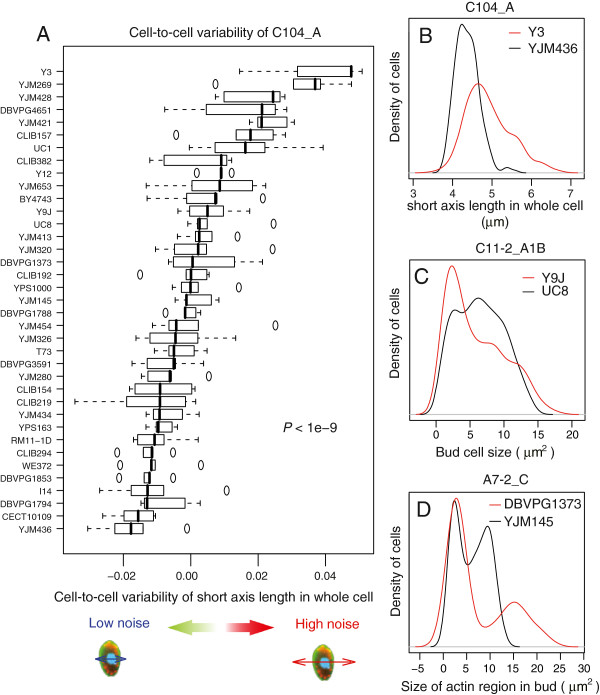
**Wild strains display different levels of noise in single-cell traits. A)** The **‘**Noise’ level of trait C104_A (short axis length of whole cell at G1) corresponds to the residuals of the lowess regression shown in Figure 5D. The boxplot represents these noise values in every strains, the *p*-value indicates significance of a strain effect as determined by Kruskal-Wallis test. **B)** Distributions of single-cell values of C104_A in strains displaying low (black, YJM436) and high (red, Y3) noise for this trait. **C)** Distributions of single-cell values of C11-2_A1B (bud size at S/G2) in strains displaying low (black, UC8) and high (red, Y9J) noise for this trait. **D)** Distributions of single-cell values of A7-2_C (size of actin region in bud at M) in strains displaying low (black, YJM145) and high (red, DBVPG1373) noise for this trait.

The fact that traits were measured on individual cells allowed us to investigate whether the level of phenotypic ‘noise’ differed between natural yeast backgrounds. Nearly half of the 501 traits reported above already estimated this intra-sample variability, since they were coefficients of variation (CVs) of measured quantities. However, these parameters sometimes varied concomitantly with the mean value of the trait considered. In agreement with previous observations made on the same type of data [8], this dependency could be positively or negatively correlated, and was not necessarily linear (Figure 5). To obtain estimates of cell-to-cell variability that were independent of mean trait values, we followed a procedure previously described that uncoupled CVs from mean by extracting residues from a lowess regression (see Methods and ref [8]). This way, 220 traits reflecting phenotypic noise *per se* were obtained for each sample. We then applied a Kruskal-Wallis test for each of these ‘noise traits’ on the null hypothesis of no strain effect. At *p* < 2.27 × 10^-4^ threshold (corresponding to *p* < 0.05 after Bonferroni correction for multiple testing), 76 noise traits were detected to be significantly affected by the strain background. This was one third of the traits considered and corresponded to variability of various cellular features: cell width, length and shape, size of actin regions within cells, bud size and orientation, and size of the bud nucleus (Additional file 6: Table S5). The trait for which cell-to-cell variation had the stronger dependence on the strain background was the short-axis length of unbudded cells (P < 10^-9^, Figure 6A-B), indicating that some backgrounds control cell width more tightly than others. Budding cells also showed traits with particularly different noise levels among strains. Bud size, for example, was more variable among Y9J cells than among UC8 cells (Figure 6C). The size of the region of bud occupied by actin was also more variable among DBVPG1373 cells than among YJM145 cells (Figure 6D). Interestingly, bud neck position (C105_A1B) also had higher cell-to-cell heterogeneity in some strains (YJM320 and YJM269) as compared to others (RM11-1D and YJM280), suggesting that all backgrounds do not control bipolar budding with equal precision.

### Phenotypic noise varies both globally and specifically

**Figure 7:**
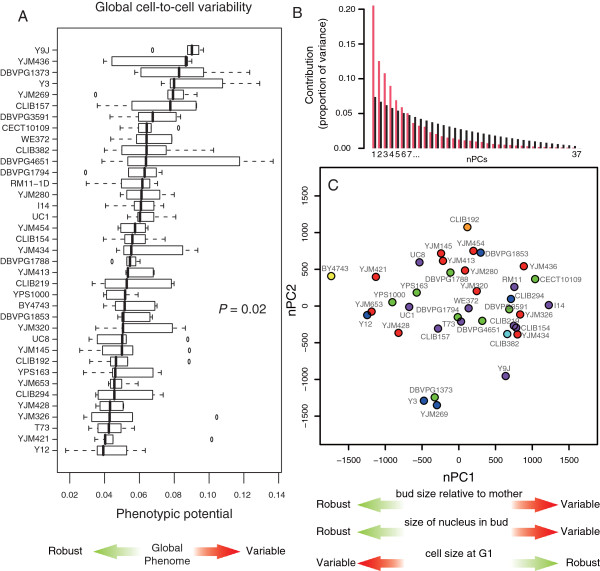
**Properties of intra-species variation of phenotypic noise. A**) Boxplot representation of phenotypic potentials of each strain. Each box represents five values, one per biological replicate. The *p*-value corresponds to the detection of a strain effect by a Kruskal-Wallis test. **B-C)** Principal component analysis performed on the 76 noise traits that significantly vary across strains. **B)** Proportion of variance explained by each component (red) as compared to random expectation (black). **C)** Coordinates of strains along the first two components. The three morphological features mentioned below the x-axis correspond to the traits best contributing to the first component.

The fact that many traits displayed strain-dependent noise raised the following question: is this variation global or specific? In the former case, one would expect to observe elevated noise of many unrelated traits in the same strains and little cell-cell variation in other strains. Alternatively, if variation is specific, a given strain may display high noise for some traits while remaining robust for other traits, and this spectrum of variability/robustness would differ between strains. To examine the first possibility, we compared strains for their phenotypic potential [8]. This value captures phenotypic noise in a broad sense, by averaging noise values from a large number of independent traits (see Methods). It was previously used on *CalMorph* datasets to detect artificial null mutations that affect general phenotypic buffering in yeast [8]. In principle, natural genetic variation may also affect the global molecular buffering of morphological traits, which would be detected by differences in phenotypic potentials among natural strains. After computing 5 independent estimates of the phenotypic potential of every strain, we observed that it significantly varied between backgrounds (Figure 7A, Kruskal-Wallis *p* = 0.02), although to a lesser extent than noise of specific traits. This shows that part of noise variation is indeed global, with strains Y9J, Y3 and DBVPG1373 showing pronounced global heterogeneities as compared to strains YJM421 and Y12. The modest statistical significance also indicates that variation is not entirely global. If it were, then the strain effect on global noise should be detected at similar or higher significance as the effect on specific noise traits, because measurement of global noise benefits from cumulated observations on various traits. This was clearly not the case, which suggests that the variation of noise is also specific.

To study this possibility, we performed a principal component analysis on the 76 noise traits that had a significant dependence on the strain background. The method is equivalent as the one presented above, except that the phenotypic values considered are now the noise of the traits instead of the trait values themselves. If noise of all traits was increased in the same strains (global variation), then the first principal component should explain most of the differences between strains, and this component should discriminate ‘noisy’ from ‘buffered’ strains. The analysis produced 7 significant components that altogether explained 71% of the variance (Figure 7B). The first component alone explained ~21% of the variance. Representing strains coordinates along these components showed that there was no obvious subgroup of strains with specific phenotypic noise values (Figure 7C). Analyzing the contribution of each trait to the principal components revealed that the first component corresponded to high variability of bud size and size of bud nucleus, but robust cell size at G1. The second component was also related to variability in bud size, whereas the third and fourth components corresponded to variability in the positioning of the dividing nucleus and variability of the size of the actin region in bud, respectively (Additional file 7: Table S6). Thus, in general, genetic backgrounds affected noise of specific sets of traits but not of all traits together. We conclude that a large fraction of cell-to-cell heterogeneity varies in a strain/trait specific manner, while another fraction varies because some strains are globally ‘noisier’ than others.

## Discussion

Morphological traits of living organisms have always fascinated evolutionary biologists since the very early days of the discipline, because they are highly informative on adaptation processes. For multicellular organisms, morphology has a direct impact on fitness, because it is tightly connected to survival (escape from predators or pathogens), reproduction, feeding, etc. This is probably less true for the morphology of yeast cells, where adaptation is guided by the shape and performances of the colony as a whole, but not of individuals. Growth ability across various environmental conditions directly reflects the fitness (propagation and adaptation) of a microorganism, whereas the shape and size of individual cells do not.

It is therefore interesting to compare the results we found here with those previously obtained on phenotypes corresponding to growth fitness. Two studies have described growth rates of various wild yeast species and strains in a large variety of environments [21,22]. This allowed the authors to define strain-strain phenomic distances that reflect fitness similarity across a broad spectrum of growth conditions. Both groups found a substantial correlation between these inter-strain distances and their degree of genetic divergence [21,22], which is consistent with accumulations of phenotypic and genotypic differences under poor selection. In addition, the growth rates of inter-strain hybrids were consistent with numerous complementations of loss of function mutations [23]. This observation, together with the fact that the yeast population structure is profoundly shaped by frequent genetic drift generated by repeated bottlenecks and expansions [24], supports the idea that mutations affecting growth rates in certain environments have accumulated over time by genetic drift. Such properties are not apparent from the morphological traits presented here: morphological similarities did not reflect relatedness in population history. Why? As mentioned above, a correlation between genetic and morphometric distances could be blurred by genomic mosaicism. However, if this were the only explanation, we would have expected to detect an association when using a subset of strains from ‘clean’ lineages, which we did not. Also, if numerous quantitative trait loci were contained in mosaic genomic portions, they would probably cause a correlation between traits and one or several genomic principal components (gPC) and we did not find any such association. Another possible explanation is the impact of environmental factors on morphological traits. We grew strains in a standardized laboratory condition that is drastically different from the natural habitats in which they normally live. This was necessary to allow for inter-strains comparisons, but the natural environment of each strain is specific and can be totally different from one strain to another. Our results are therefore not in contradiction with previously reported correlations based on growth in various environments. Another possible interpretation is that morphological variation may have fewer degrees of freedom than growth fitness across various environments. The topological organization of cells is limited by physical constraints and highly conserved cellular mechanisms, whereas growth efficiency is guided by metabolic activities and stress responses that benefit from flexible and complex molecular networks. These constraints on cellular organization and morphology likely apply across many environmental conditions, preventing accumulation of relevant loss of function mutations in isolated subpopulations. Extending our study to the morphological profiling of diploid hybrids would be interesting in this regard: additivity would suggest gradual drift of cellular regulations whereas non-additivity would imply more discrete phenotypic changes possibly emerging from loss of function mutations.

Our results provide an estimate of the natural variation of phenotypic noise among natural populations. Since our experimental design included enough biological replicates of sufficient sample size, we could test whether cell-to-cell heterogeneities were more pronounced in some clonal populations as compared to others. We obtained three major conclusions. First, one third of cellular traits (76 out of 220) had noise levels that were significantly affected by the strain background. This remarkable proportion shows that many cellular regulations are not equally buffered in every strain. Secondly, when pooling noise values of unrelated traits into a single metric (phenotypic potential), we found that some backgrounds were generally ‘noisy’ as compared to others. Importantly, this variation in general noise was not associated with relatedness of the strains. For example, strains Y9J, YJM269 and Y3 all had global noise but represented various branches of the genetic tree. Finally, decomposing traits with varying noise levels showed a substantial specificity regarding which traits were noisy in which strains. In other words, phenotypic noise did not vary only because some strains were globally noisy but also because some strains were noisy for specific subsets of traits. This observation complements previous reports made on artificial null mutations. Levy and Siegal computed phenotypic potentials from *CalMorph* morphological profiles of systematic gene deletion mutants [8]. They observed that high global noise was associated with mutations targeting genes that 1) were highly connected in networks of protein-protein or synthetic lethality interactions and 2) were essential for efficient cell growth. When occurring in the wild, such dramatic loss-of-function mutations are probably counter-selected, for they affect numerous cellular regulations and likely reduce fitness in a wide range of environmental conditions. The results presented here are therefore important as they show the properties of noise variation across *natural* genetic contexts. Global noise significantly differed among strains. This may result from DNA polymorphisms targeting capacitor genes, by producing more subtle changes of activity than full inactivation. Alternatively, it may result from the accumulation of mutations on various regulatory pathways, each contributing to a reduced buffering. However, the pattern of noise variation that we observed clearly tended to be specific. This is particularly apparent in the principal component analysis: the analysis did not discriminate any subgroup of strains with particularly high noise levels, and the first component obtained was made up of two traits with high noise (size of bud and of its nucleus) and one trait with low noise (cell size at G1). There is no straightforward interpretation to why these noises appear anticorrelated, but this illustrates that noise of individual traits vary rather independently from one another. This independence probably results from mutations affecting specific molecular pathways. Dissecting the molecular sources of noise in cellular traits would be very informative. This may be achieved by treating noise as a complex trait in a quantitative genetics design, as was done for the regulation of gene expression [10].

A fascinating question is whether evolutionary forces directly modulate phenotypic noise levels. A simulation by Wang and Zhang showed that global gene expression noise in metabolic pathways dramatically affects fitness and is likely counter-selected [25]. This study also suggests that noise can slow the rate of fixation of beneficial mutations. Nonetheless, in the context of fluctuating environments, maintaining intra-clonal diversity may be advantageous and elevated noise itself may be selected for [7,26]. In other words, noise may simply result from a relaxed buffering when some traits no longer need to be precisely controlled, or it may result from adaptive strategies that bet on long-term survival through environmental perturbations (bet hedging). Such strategies were found to happen in yeast when individual cells challenged by heat-shock were monitored [27]. Bet hedging may therefore happen in the wild to maintain elevated noise. Our results do not prove that this is the case, but they add two very important factual observations: noise levels do differ between natural subpopulations, and this variation happens rather independently from one trait to another. Thus, microevolution may take place on these contemporary genotypes by selecting for or against the ones that maintain individuals with different physiological properties than the bulk of the clonal population. In this respect, increasing noise of only a few traits in some backgrounds is likely advantageous. This modularity may confer trait-specific adaptive potential without affecting global robustness. Now that we identified which wild backgrounds displayed elevated noise for some traits, it will be interesting to test whether they confer fitness advantages in fluctuating environments or during exposure to environmental catastrophes. This would exemplify how natural genotypes can favor bet-hedging strategies.

## Conclusions

By profiling numerous traits of thousands of individual cells from different wild genetic backgrounds of yeast, we found abundant intra-species variation of cellular morphology and internal organization. These phenotypic differences did not reflect the population history of the species. Importantly, our results show that phenotypic noise does vary between natural backgrounds. Thus, microevolution may take place in the wild to fix or discard genotypes conferring elevated phenotypic noise.

## Methods

### Strains

Strains used are listed in Additional file 1: Table S1.

### Morphological profiling

Yeast cells were grown in synthetic growth medium [SD; 0.67% yeast nitrogen base without amino acid (Difco), and 2% glucose (Wako Chemicals)], with appropriate amino acid and base supplements. The final concentration of each amino acid supplement was 20 μg/ml for adenine, uracil, histidine, methionine and 30 μg/ml for leucine. Cells were cultured in the 20 ml liquid SD medium at 30°C to logarithmic-phase. Cell fixation, staining and image acquisition were performed as described previously [13]. At least 200 cells were captured in a set of acquired images from an independent cell culture. A total of 185 sets of images were acquired from five replicated experiments on each of the 37 strains. The image sets were processed with the *CalMorph* software (version 1.3) as described previously [13].

### Statistical tests for strain effects

All statistical analyses were done using R (http://www.r-project.org). A Kruskal-Wallis rank sum test was performed for every trait against the null hypothesis of no strain effect. The dataset contained, for each trait, 5 independent values per strain, across 37 strains. We compared the observed values of the *K* statistics with an empirical null distribution obtained by running the test 1,000 times on permuted datasets. At each permutation, all 185 traits values were re-attributed to strains, so that each strain was associated with 5 randomly picked values. On average across these permutations, only 4.15 traits showed *K* > 56, whereas this threshold was reached for 440 traits when using the actual dataset. We therefore used this list of 440 significant traits corresponding to FDR = 0.01.

### Principal component analysis

We first transformed the raw dataset of 185 × 501 trait values into sums of ranks: for each trait, every strain was assigned the sum of its 5 ranks as previously described [28]. This resulted in a 37 × 501 phenotypic matrix on which we applied the prcomp() function from R using default parameter values.

### Statistical test for effect of ecological or geographical origins

For each trait, a possible association to the ecological or geographical origin of the strains was tested as follows. For each strain, the trait values across the 5 replicates were averaged. We then applied a Kruskal-Wallis rank sum test on the factor of interest (ecology or geography). The lowest *p*-values obtained across all traits were 0.01 and 0.001 for ecological and geographical origin, respectively. Given the multiplicity of the test (501 traits), we concluded that no significant association could be claimed.

### Hierarchical cluster analysis

To detect groups of strains sharing similar morphology, hierarchical clustering was performed by the average linkage using the R package *pvclust*[19]. Using the principal component scores from PC1 to PC28 covering more than 97% of the cumulative contribution ratio, the morphological dissimilarity between any pair of the 37 strains was computed as an angle as previously described [29]. Clusters were detected at *P* > 0.95 by the multi-scale bootstrap technique with 10000 iterations [19].

### Linear discriminant analysis

To assess the morphological features of the three strain groups I, II and III, we performed a linear discriminant analysis (LDA) using the *lda()* function of the R package *MASS*. To ensure discrimination, 268 of 501 parameters were selected by the Kruskal-Wallis test at *p* < 0.01 after Bonferroni correction. With the class labels determined by the cluster analysis, LDA was applied on the 268 rank-sumed parameter values of the 37 strains, and the predicted classes of each strain by the LDA were completely matched to the class labels from the cluster analysis. To select the parameters discriminating the classes, the interior angles between the eigenvector of each parameter and the center vector of the strains of each class projected on the three dimensional linear discriminant space were computed as the contribution score, and were compared with the maximum angle in the strains of each class. The maximum angles among the strains of the class I, II and III were 30.35 degrees (DBVPG1794), 20.50 degrees (YPS1000) and 18.30 degrees (YJM454), respectively. Of the 268 parameters, 39, 9 and 19 parameters scored below the maximum angle of the strains of the class I, II and III, respectively (Additional file 5: Table S4). The projections of the strains on the linear discriminant space were mapped onto the center vectors to calculate a representative score for each class, and the correlation coefficient of the rank-sum values of 268 parameters to the representative scores were computed to select a representative parameter for the cell morphology of each class (Additional file 5: Table S4). From Additional file 5: Table S4, the parameters of high correlation coefficient were selected as the parameters representing the cell morphology of G1, S/G2 and M in each class (Additional file 3: Figure S4), and were summarized in Figure 3B.

### Correlation analysis between the genetic distances and phenotypic similarities

Phenotypic similarity between any two strains was computed as the Pearson’s product–moment correlation coefficient of the strains coordinates along the first 28 pPCs. Genetic distances were those previously described [14]. Figure 4A shows these phenotypic similarities (y-axis) and genetic distances (x-axis) for 666 pairs of strains. The Spearman rank correlation coefficient between these two measures was −0.08. To see if a correlation was better detected in clean non-mosaic lineages, we selected 16 strains (Additional file 1: Table S1) belonging to a cluster of wine strains and previously shown to have a lineage that was monomorphic for the majority of segregating sites. These isolates exhibit the same phylogenetic relationship across their entire genome and a previous analysis with STRUCTURE showed that the estimated ancestry proportion is greater than 0.9 for all these 16 strains [14]. We therefore considered them to come from non-mosaic lineages and we re-calculated the correlation coefficient between genetic and phenotypic distances as above but using data from these 16 strains only. The correlation coefficient obtained was −0.05, showing no improvement.

### Correlation analysis between the genetic and phenotypic population structures

To test for the correlation between the genetic population structure and the morphological features among the 37 strains, we computed Spearman's rank correlation coefficients between the principal component scores of the genotypes (gPC) and the phenotypes (pPC). The principal components of the genotypic variance was obtained by applying the prcomp() function of R on the SNP data of Schacherer et al. [14]. Spearman’s rank correlation coefficients were computed among all pairwise combinations between the 37 gPCs and the 37 pPCs. The correlation coefficients were distributed between −0.555 and 0.547. A permutation test showed that none of these correlation was significant at FDR = 0.05.

### Statistical tests on cell-to-cell variations

Of the 501 parameters computed by *CalMorph*, 220 correspond to single-cell measures that were averaged across the sample. Another 220 parameters are the coefficients of variation (CV) of the same measures, and the remaining 61 parameters reflect other properties of the sample, such as the fraction of cells at a given division stage. An example of an average trait is parameter D182_A, which is the mean value of the nuclear axis ratio acquired from all cells in G1 of a sample. This parameter is coupled to parameter DCV182_A, which is the coefficient of variation of this trait across the same cells. This way, the entire set of parameters summarizes both mean and variance values of morphological traits. Intuitively, coefficients of variation provide solid estimates of cell-to-cell heterogeneities, as they are free of dimension. However, CV values were shown to depend highly on mean trait values, and this dependence is known to be non-linear on *CalMorph* outputs [8]. We therefore used a method proposed by Levy & Siegal [8] to uncouple this dependency, by applying a lowess regression to condition CV on mean values. This was done using the lowess() function of R with a smoother span of 0.4. Examples of fits are shown on Figure 5. We then defined ‘noise traits’ as the residuals (i.e. observed - predicted values) of the model. This way, 220 noise traits were computed on five independent samples of each strain. For every noise trait, a Kruskal-Wallis test was applied on the null hypothesis of no strain effect. 46 and 76 noise traits proved significant at *p* < 0.01 and *p* < 0.05, respectively, after Bonferroni correction (Additional file 6: Table S5).

To estimate global phenotypic noise (instead of trait-specific noise), we used the *phenotypic potentials* as defined by Levy and Siegal [8]. To compute these estimates, a list of non-redundant traits must be selected. This dimension reduction is important to avoid calling ‘global’ an observation that would in fact be specific to a set of traits that are highly correlated (redundant measurements of the same cellular property). To do this in an unbiased way, we used the list of 70 traits validated by Levy and Siegal who performed a Partitioning Around the Medoids (PAM) clustering analysis on a previously generated *CalMorph* dataset [13]. This dataset was larger than the one produced here, and it included extreme genetic perturbations. It therefore offered a better framework to infer trait-to-trait independence. Using this list of 70 medoid traits, we reduced our matrix of noise traits from 185 × 220 to 185 × 70 values. We then computed the phenotypic potential of each sample as the mean of its 35 highest noise values. This way, 5 independent estimates of phenotypic potentials were obtained per strain, and a Kruskal-Wallis test was applied to test for a strain effect on these values.

### Principal component analysis on noise traits

We considered only the 76 noise traits that were significantly affected by the strain background. We first transformed the dataset of 185 × 76 noise trait values into sums of ranks: for each noise trait, every strain was assigned the sum of its 5 ranks as previously described [28]. This resulted in a 37 × 76 matrix on which we applied the prcomp() function from R using default parameter values. Then the principal component (PC) loadings were calculated, where the PC loading is statistically equivalent to the correlation coefficients (R) between each of the 7 first noise principal components (nPC) and each of the 76 noise traits (532 combinations). To test for significant correlation values, we examined if *T* = R x [ (n-2) / (1-R^2^) ]^1/2^, where *n* = 37 is the sample size, significantly deviated from the *t*-distribution with *n* - 2 degrees of freedom. We applied a Bonferroni correction to retain only those with nominal *p*-value lower than 0.05/532, which are listed in Additional file 7: Table S6.

### Availability of supporting data

Raw images and datasets are freely available at http://sunlight.k.u-tokyo.ac.jp/wild37noise/index.html.

## Competing interests

The authors declare that they have no competing interests.

## Authors’ contributions

GY: Conceived and designed the study, analyzed the data, interpreted the results, developed analysis codes, wrote the paper. SO: Analyzed the data, interpreted the results, developed analysis codes and made figures of the paper. SN: Managed experiments. YI: Performed the experiments. SF: Interpreted the results. JS: Contributed strains and interpreted the results. YO: Conceived and designed the study, directed experiments and analyses, interpreted the results. All authors read and approved the final manuscript.

## Supplementary Material

Additional file 1: Table S1 List of strains used in this study. Click here for file

Additional file 2: Table S2 List of 440 traits with significant inter-strain variation at FDR 1%. Click here for file

Additional file 3: Figure S1 Cumulative proportion of variance of the principal component analysis for the phenotypes. Black and grey bars indicate the proportion of variance (left axis) explained by the pPCs without randomization and after randomization, respectively. Red circles and rectangles indicate the cumulative proportion of variance (right axis) explained by the pPCs without randomization and after randomization, respectively. The horizontal dashed red line indicates 97% of the cumulative proportion of variance. **Figure S2.** Principal component analysis of *S. cerevisiae* morphological variation**.** Dots represent strains by their coordinates along principal components pPC3 and pPC4, from the same PCA analysis as in Figure 2A. **Figure S3.** Heatmap of the rank-sum values of the parameters contributing to discriminate strain classes I, II and III by LDA**.** The dendrogram and strain labels at the top are the same as in Figure 3A. Three heatmaps indicate the rank-sum values of the representative parameters for the class I, II and III from top to bottom, respectively. Red, black, and green, indicate high, middle and low values, respectively. Pink, greenyellow and lightorange rectangles on the heatmap indicate the class I, II and III of strains, respectively. **Figure S4.** Strains distribution along the parameters representing the morphological features of each class**.** Pink, greenyellow, lightorange and black circles indicate strains of classes I, II, III and others, respectively. Red frames indicate scatter plots of the distribution of the 37 strains on the representative parameters for each class. **A**) Parameters representative of Class I. **B**) Parameters representative of Class II. **C**) Parameters representative of Class III. **Figure S5.** Principal component analysis of *S. cerevisiae* genetic variation. A boolean matrix of the single nucleotide polymorphisms (Schacherer et al. [19]) was used for principal component analysis. **A**) Strains are represented by their coordinates along the first two principal components, using the same colors as in Figure 1A. **B**) Cumulative proportion of variance**.** Grey bars indicate the proportion of variance (left axis) in the gPCs. Red circles indicate the cumulative proportion of variance (right axis) in the gPCs. Horizontal broken red line indicates 0.45 of the cumulative proportion of variance. Click here for file

Additional file 4: Table S3 Parameters contributing to the first four phenotypic principal components. Click here for file

Additional file 5: Table S4 Parameters contributing to phenotypic classes I, II and III, as determined by Linear Discriminant Analysis. Click here for file

Additional file 6: Table S5 List of 76 noise traits with significant inter-strain variation. Click here for file

Additional file 7: Table S6 Noise traits contributing to the first six noise principal components. Click here for file

## References

[B1] Eldar A Chary VK Xenopoulos P Fontes ME Loson OC Dworkin J Piggot PJ Elowitz MB Partial penetrance facilitates developmental evolution in bacteria Nature 2009 460 510 514 19578359 10.1038/nature08150 PMC2716064

[B2] Raj A Rifkin SA Andersen E van Oudenaarden A Variability in gene expression underlies incomplete penetrance Nature 2010 463 913 918 10.1038/nature08781 20164922 PMC2836165

[B3] Balaban NQ Merrin J Chait R Kowalik L Leibler S Bacterial persistence as a phenotypic switch Science 2004 305 1622 1625 10.1126/science.1099390 15308767

[B4] Beaumont HJ Gallie J Kost C Ferguson GC Rainey PB Experimental evolution of bet hedging Nature 2009 462 90 93 10.1038/nature08504 19890329

[B5] Stomp M van Dijk MA van Overzee HM Wortel MT Sigon CA Egas M Hoogveld H Gons HJ Huisman J The timescale of phenotypic plasticity and its impact on competition in fluctuating environments Am Nat 2008 172 169 185 10.1086/591680 18828745

[B6] Kuwahara H Soyer OS Bistability in feedback circuits as a byproduct of evolution of evolvability Mol Syst Biol 2012 8 564 22252387 10.1038/msb.2011.98 PMC3296359

[B7] Zhang Z Qian W Zhang J Positive selection for elevated gene expression noise in yeast Mol Syst Biol 2009 5 299 19690568 10.1038/msb.2009.58 PMC2736655

[B8] Levy SF Siegal ML Network hubs buffer environmental variation in *Saccharomyces cerevisiae* PLoS Biol 2008 6 e264 10.1371/journal.pbio.0060264 18986213 PMC2577700

[B9] Carter AJ Houle D Artificial selection reveals heritable variation for developmental instability Evolution 2011 65 3558 3564 10.1111/j.1558-5646.2011.01393.x 22133225

[B10] Ansel J Bottin H Rodriguez-Beltran C Damon C Nagarajan M Fehrmann S Francois J Yvert G Cell-to-cell stochastic variation in gene expression is a complex genetic trait PLoS Genet 2008 4 e1000049 10.1371/journal.pgen.1000049 18404214 PMC2289839

[B11] Becskei A Serrano L Engineering stability in gene networks by autoregulation Nature 2000 405 590 593 10.1038/35014651 10850721

[B12] Jones EW Pringle JR 1992 Broach JR: The molecular and cellular biology of the yeast Saccharomyces. Cold Spring Harbor Laboratory Press

[B13] Ohya Y Sese J Yukawa M Sano F Nakatani Y Saito TL Saka A Fukuda T Ishihara S Oka S High-dimensional and large-scale phenotyping of yeast mutants Proc Natl Acad Sci USA 2005 102 19015 19020 10.1073/pnas.0509436102 16365294 PMC1316885

[B14] Schacherer J Shapiro JA Ruderfer DM Kruglyak L Comprehensive polymorphism survey elucidates population structure of *Saccharomyces cerevisiae* Nature 2009 458 342 345 10.1038/nature07670 19212320 PMC2782482

[B15] Nogami S Ohya Y Yvert G Genetic complexity and quantitative trait loci mapping of yeast morphological traits PLoS Genet 2007 3 e31 10.1371/journal.pgen.0030031 17319748 PMC1802830

[B16] Zahner JE Harkins HA Pringle JR Genetic analysis of the bipolar pattern of bud site selection in the yeast *Saccharomyces cerevisiae* Mol Cell Biol 1996 16 1857 1870 8657162 10.1128/mcb.16.4.1857 PMC231173

[B17] Sheu YJ Barral Y Snyder M Polarized growth controls cell shape and bipolar bud site selection in *Saccharomyces cerevisiae* Mol Cell Biol 2000 20 5235 5247 10.1128/MCB.20.14.5235-5247.2000 10866679 PMC85972

[B18] Bhatta H Goldys EM Quantitative characterization of different strains of Saccharomyces yeast by analysis of fluorescence microscopy images of cell populations J Microbiol Methods 2009 77 77 84 10.1016/j.mimet.2009.01.011 19318060

[B19] Suzuki R Shimodaira H Pvclust: an R package for assessing the uncertainty in hierarchical clustering Bioinformatics 2006 22 1540 1542 10.1093/bioinformatics/btl117 16595560

[B20] Shimodaira H An approximately unbiased test of phylogenetic tree selection Syst Biol 2002 51 492 508 10.1080/10635150290069913 12079646

[B21] Warringer J Zorgo E Cubillos FA Zia A Gjuvsland A Simpson JT Forsmark A Durbin R Omholt SW Louis EJ Trait variation in yeast is defined by population history PLoS Genet 2011 7 e1002111 10.1371/journal.pgen.1002111 21698134 PMC3116910

[B22] Jarosz DF Lindquist S Hsp90 and environmental stress transform the adaptive value of natural genetic variation Science 2010 330 1820 1824 10.1126/science.1195487 21205668 PMC3260023

[B23] Zorgo E Gjuvsland A Cubillos FA Louis EJ Liti G Blomberg A Omholt SW Warringer J Life history shapes trait heredity by promoting accumulation of loss-of-function alleles in yeast Mol Biol Evol 2012 29 1781 1789 10.1093/molbev/mss019 22319169

[B24] Dujon B Yeast evolutionary genomics Nat Rev Genet 2010 11 512 524 20559329 10.1038/nrg2811

[B25] Wang Z Zhang J Impact of gene expression noise on organismal fitness and the efficacy of natural selection Proc Natl Acad Sci USA 2011 108 E67 E76 10.1073/pnas.1100059108 21464323 PMC3080991

[B26] Acar M Mettetal JT van Oudenaarden A Stochastic switching as a survival strategy in fluctuating environments Nat Genet 2008 40 471 475 10.1038/ng.110 18362885

[B27] Levy SF Ziv N Siegal ML Bet hedging in yeast by heterogeneous, age-correlated expression of a stress protectant PLoS Biol 2012 10 e1001325 10.1371/journal.pbio.1001325 22589700 PMC3348152

[B28] Okada H Abe M Asakawa-Minemura M Hirata A Qadota H Morishita K Ohnuki S Nogami S Ohya Y Multiple functional domains of the yeast l,3-beta-glucan synthase subunit Fks1p revealed by quantitative phenotypic analysis of temperature-sensitive mutants Genetics 2010 184 1013 1024 10.1534/genetics.109.109892 20124029 PMC2865904

[B29] Ohnuki S Nogami S Kanai H Hirata D Nakatani Y Morishita S Ohya Y Diversity of Ca2^+^-induced morphology revealed by morphological phenotyping of Ca^2+^-sensitive mutants of *Saccharomyces cerevisiae* Eukaryot Cell 2007 6 817 830 10.1128/EC.00012-07 17351076 PMC1899241

