# Supplementary material for: Single-cell phenomics reveals intra-species variation of phenotypic noise in yeast
Source: BMC Syst Biol. 2013 Jul 3;7:54. doi: 10.1186/1752-0509-7-54 (PMC3711934; doi:10.1186/1752-0509-7-54)
Supplement: Additional file 1: Table S1 — List of strains used in this study. [file 1752-0509-7-54-S1.doc]

**Table S1.** Description of *S. cerevisiae* strains studied

| **Strains^#^** | **Sources** | | **Location** | **Number**  **of SNPs*** |
| --- | --- | --- | --- | --- |
| CLIB192 | | Baker | France | 11850 |
|  | |  |  |  |
| CLIB382 | | Beer | Japan | 26779 |
|  | |  |  |  |
| YJM145 | | Clinical. AIDS patient | United States | 39910 |
| YJM280 | | Clinical. peritoneal fluid | United States | 37393 |
| YJM320 | | Clinical. blood | United States | 34089 |
| YJM326 | | Clinical. unknown | United States | 34841 |
| YJM413 | | Clinical. blood | United States | 35986 |
| YJM421 | | Clinical. ascites fluid | United States | 38058 |
| YJM428 | | Clinical. paracenteis fluid | United States | 32784 |
| YJM434 | | Clinical. unknown | Europe | 30171 |
| YJM436 | | Clinical. mouth | Europe | 29199 |
| YJM454 | | Clinical. blood | United States | 39049 |
| YJM653 | | Clinical. brochoalveolar lavage | United States | 34915 |
|  | |  |  |  |
| CECT10109 | | Prickly pear | Spain | 33325 |
| DBVPG1373 | | Soil | Netherlands | 30931 |
| DBVPG1788 | | Soil | Finland | 26132 |
| DBVPG1794 | | Soil | Finland | 31806 |
| DBVPG3591 | | Cocoa beans | Unknown | 25048 |
| DBVPG4651 | | Tuber Magnatum | Italy | 30602 |
| YPS1000 | | Oak exudates | United States | 44250 |
| YPS163 | | Oak exudates | United States | 39189 |
|  | |  |  |  |
| CLIB294 | | Distillery | France | 26877 |
| DBVPG1853 | | White Tecc | Ethiopia | 38585 |
| Y12 | | Palm Wine | Ivory Coast | 38304 |
| Y3 | | Palm Wine | Africa | 40996 |
| YJM269 | | Apple juice | Unknown | 41336 |
|  | |  |  |  |
| BY4743 | | Laboratory |  | NA |
|  | |  |  |  |
| CLIB154 | | Wine | Russia | 22942 |
| CLIB157 | | Wine | Spain | 26612 |
| CLIB219 | | Wine | Russia | 47840 |
| I14 | | Vineyard soil | Italy | 27442 |
| RM11 | | Wine | California | 29508 |
| T73 | | Wine | Spain | 24636 |
| UC1 | | Wine | France | 26077 |
| UC8 | | Wine | South Africa | 29900 |
| WE372 | | Wine | South Africa | 28510 |
| Y9J | | Wine | Japan | 30678 |

* Number of calls detected by Schacherer et al. 2009.

# Names of the 16 strains belonging to non-mosaic lineages are underlined (see methods).
