# Supplementary material for: Single-cell phenomics reveals intra-species variation of phenotypic noise in yeast
Source: BMC Syst Biol. 2013 Jul 3;7:54. doi: 10.1186/1752-0509-7-54 (PMC3711934; doi:10.1186/1752-0509-7-54)
Supplement: Additional file 2: Table S2 — List of 440 traits with significant inter-strain variation at FDR 1%. [file 1752-0509-7-54-S2.doc]

**Table S2.** List of traits showing significant inter-strain variability

| **No.** | **ID** | ***K*** | **Description** |
| --- | --- | --- | --- |
| 1 | C115_A1B | 174.70 | Mother_axis_ratio |
| 2 | C115_A | 171.79 | Whole_cell_axis_ratio |
| 3 | C115_C | 169.81 | Mother_axis_ratio |
| 4 | C114_C | 163.52 | Bud_axis_ratio |
| 5 | C128_A1B | 163.04 | Distance_between_middle_point_of_neck_and_mother_hip |
| 6 | C103_A1B | 161.90 | Long_axis_length_in_mother |
| 7 | C112_A1B | 159.06 | Distance_between_middle_point_of_neck_and_mother_center |
| 8 | C103_C | 157.50 | Long_axis_length_in_mother |
| 9 | C128_C | 155.77 | Distance_between_middle_point_of_neck_and_mother_hip |
| 10 | C109_A1B | 153.68 | Neck_width |
| 11 | CCV115_A1B | 153.62 | Coefficient_of_variation_of_C115_A1B |
| 12 | C112_C | 153.00 | Distance_between_middle_point_of_neck_and_mother_center |
| 13 | C114_A1B | 152.61 | Bud_axis_ratio |
| 14 | C103_A | 152.31 | Long_axis_length_in_whole_cell |
| 15 | D129_A1B | 152.04 | Distance_between_nuclear_brightest_point_and_mother_tip |
| 16 | C12-1_A1B | 150.87 | Mother_cell_outline_length |
| 17 | C111_A1B | 150.68 | Distance_between_bud_tip_and_mother_short_axis_extension |
| 18 | D104_A1B | 150.04 | Distance_between_nuclear_gravity_center_and_mother_tip |
| 19 | D185_C | 148.42 | Total_length_of_two_straight_segments_D11-1C4-1_and_D11-2C4-1 |
| 20 | C105_A1B | 147.66 | Neck_position |
| 21 | D186_C | 147.07 | Total_length_of_two_straight_segments_D12-1C4-1_and_D12-2C4-1 |
| 22 | D142_A1B | 146.42 | Distance_between_nuclear_brightest_point_and_mother_hip |
| 23 | C12-1_C | 145.71 | Mother_cell_outline_length |
| 24 | C13_A1B | 145.67 | Mother_cell_fitness_for_ellipse |
| 25 | C102_C | 143.57 | Whole_cell_outline_length |
| 26 | C11-1_A1B | 143.15 | Mother_cell_size |
| 27 | D126_A1B | 142.73 | Distance_between_nuclear_gravity_center_and_mother_hip |
| 28 | D102_A | 142.45 | Distance_between_nuclear_gravity_center_and_mother_tip |
| 29 | D127_A | 141.42 | Distance_between_nuclear_brightest_point_and_cell_tip |
| 30 | C109_C | 141.10 | Neck_width |
| 31 | D154_A1B | 140.64 | Angle_between_C1D1-1_and_C1C1-2 |
| 32 | C12-1_A | 140.63 | Whole_cell_outline_length |
| 33 | C104_A | 140.58 | Short_axis_length_in_whole_cell |
| 34 | C104_A1B | 140.21 | Short_axis_length_in_mother |
| 35 | D155_A1B | 138.99 | Angle_between_C1D2-1_and_C1C1-2 |
| 36 | C11-1_A | 138.74 | Whole_cell_size |
| 37 | C101_C | 138.31 | Whole_cell_size |
| 38 | C126_A | 137.90 | Brightness_difference_of_cell_wall |
| 39 | D132_A1B | 137.70 | Distance_between_nuclear_brightest_point_and_middle_point_of_neck |
| 40 | C11-1_C | 136.63 | Mother_cell_size |
| 41 | CCV111_A1B | 136.25 | Coefficient_of_variation_of_C111_A1B |
| 42 | CCV115_A | 135.48 | Coefficient_of_variation_of_C115_A |
| 43 | C126_A1B | 134.94 | Brightness_difference_of_cell_wall |
| 44 | C104_C | 134.92 | Short_axis_length_in_mother |
| 45 | D170_A1B | 134.28 | Angle_between_C4-1D2-1_and_C4-1C1 |
| 46 | A105 | 133.86 | actin_a_ratio |
| 47 | C107_C | 133.38 | Long_axis_length_in_bud |
| 48 | C101_A1B | 132.88 | Whole_cell_size |
| 49 | D204 | 132.85 | nuclear_E_ratio |
| 50 | C102_A1B | 132.52 | Whole_cell_outline_length |
| 51 | C108_C | 132.04 | Short_axis_length_in_bud |
| 52 | D145_A1B | 131.78 | Distance_between_nuclear_outline_point_D7_and_mother_hip |
| 53 | A114 | 131.59 | actin_a_ratio_to_no_bud_cells |
| 54 | A115 | 131.59 | actin_b_ratio_to_no_bud_cells |
| 55 | A9_A1B | 131.51 | Proportion_of_actin_region_at_neck |
| 56 | A101_A | 131.34 | Actin_region_ratio_in_whole_cell |
| 57 | C111_C | 130.36 | Distance_between_bud_tip_and_mother_short_axis_extension |
| 58 | A106_A | 130.03 | Actin_b_ratio |
| 59 | C12-2_C | 129.95 | Bud_cell_outline_length |
| 60 | C110_A1B | 129.94 | Distance_between_bud_tip_and_mother_long_axis_extension |
| 61 | A9_C | 129.91 | Proportion_of_actin_region_at_neck |
| 62 | C11-2_C | 129.45 | Bud_cell_size |
| 63 | D110_A1B | 129.16 | Distance_between_nuclear_gravity_center_and_middle_point_of_neck |
| 64 | A105_A | 129.08 | Actin_a_ratio |
| 65 | D135_A | 128.79 | Distance_between_nuclear_brightest_point_and_cell_center |
| 66 | C108_A1B | 128.68 | Short_axis_length_in_bud |
| 67 | C106_A1B | 127.92 | Bud_direction |
| 68 | C126_C | 127.80 | Brightness_difference_of_cell_wall |
| 69 | D148_A1B | 127.74 | Relative_distance_of_nuclear_brightest_point_to_mother_center |
| 70 | D169_A1B | 127.21 | Angle_between_C4-1D1-1_and_C4-1C1 |
| 71 | C13_C | 126.78 | Mother_cell_fitness_for_ellipse |
| 72 | D203 | 126.41 | nuclear_D_ratio |
| 73 | D148_A | 125.77 | Relative_distance_of_nuclear_brightest_point_to_cell_center |
| 74 | D134_C | 125.05 | Distance_between_two_nuclear_brightest_points_through_middle_point_of_neck |
| 75 | CCV104_A | 124.72 | Coefficient_of_variation_of_C104_A |
| 76 | CCV114_C | 124.30 | Coefficient_of_variation_of_C114_C |
| 77 | D116_C | 123.52 | Distance_between_two_nuclear_gravity_centers_through_middle_point_of_neck |
| 78 | D125_C | 123.17 | Distance_between_nuclear_gravity_center_in_mother_and_mother_hip |
| 79 | D143_A1B | 122.83 | Distance_between_nuclear_outline_point_D6-1_and_middle_point_of_neck |
| 80 | ACV9_A1B | 122.34 | Coefficient_of_variation_of_A9_A1B |
| 81 | C118_C | 121.75 | Cell_size_ratio |
| 82 | D103_C | 121.71 | Distance_between_nuclear_gravity_center_in_mother_and_mother_tip |
| 83 | CCV11-1_A | 121.69 | Coefficient_of_variation_of_C11-1_A |
| 84 | D14-3_C | 121.20 | Nuclear_size_in_whole_cell |
| 85 | C13_A | 120.00 | Whole_cell_fitness_for_ellipse |
| 86 | C116_A1B | 119.56 | Axis_ratio_ratio |
| 87 | A7-1_A | 119.11 | Size_of_actin_region |
| 88 | A108 | 119.11 | actin_d_iso_ratio |
| 89 | D206 | 118.42 | nuclear_A_ratio_to_no_bud_cells |
| 90 | D141_C | 117.42 | Distance_between_nuclear_brightest_point_in_mother_and_mother_hip |
| 91 | D181_A1B | 117.29 | Nuclear_minimum_radius |
| 92 | A111 | 117.20 | actin_ae_ratio |
| 93 | C117_C | 116.68 | Cell_outline_ratio |
| 94 | C105_C | 116.22 | Neck_position |
| 95 | DCV154_A1B | 116.00 | Coefficient_of_variation_of_D154_A1B |
| 96 | C113_C | 115.90 | Distance_between_bud_tip_and_mother_long_axis_through_middle_point_of_neck |
| 97 | D108_C | 115.63 | Distance_between_nuclear_gravity_center_in_mother_and_middle_point_of_neck |
| 98 | D118_A1B | 115.00 | Distance_between_nuclear_gravity_center_and_mother_center |
| 99 | D14-3_A1B | 114.81 | Nuclear_size |
| 100 | D130_C | 114.73 | Distance_between_nuclear_brightest_point_in_mother_and_middle_point_of_neck |
| 101 | DCV155_A1B | 114.25 | Coefficient_of_variation_of_D155_A1B |
| 102 | CCV12-1_A | 113.96 | Coefficient_of_variation_of_C12-1_A |
| 103 | ACV8-1_A1B | 113.49 | Coefficient_of_variation_of_A8-1_A1B |
| 104 | A8-2_C | 113.49 | Total_brightness_of_actin_region_in_bud |
| 105 | C118_A1B | 113.38 | Cell_size_ratio |
| 106 | D145_C | 112.83 | Distance_between_nuclear_outline_point_D7_in_mother_and_mother_hip |
| 107 | CCV116_C | 112.42 | Coefficient_of_variation_of_C116_C |
| 108 | D136_A1B | 112.39 | Distance_between_nuclear_brightest_point_and_mother_center |
| 109 | D128_C | 111.63 | Distance_between_nuclear_brightest_point_in_mother_and_mother_tip |
| 110 | C117_A1B | 111.56 | Cell_outline_ratio |
| 111 | D147_A1B | 111.01 | Relative_distance_of_nuclear_gravity_center_to_mother_center |
| 112 | D199 | 110.96 | nuclear_A_ratio |
| 113 | D200 | 110.46 | nuclear_A1_ratio |
| 114 | D107_A1B | 109.69 | Ratio_of_D104_to_C103 |
| 115 | C119 | 109.14 | no_bud_ratio |
| 116 | ACV7-1_A1B | 109.10 | Coefficient_of_variation_of_A7-1_A1B |
| 117 | D210 | 109.01 | nuclear_A_ratio_to_nuclear_AA1BC_cells |
| 118 | A107_A1B | 108.52 | Actin_c_api_ratio |
| 119 | D131_C | 108.35 | Distance_between_nuclear_brightest_point_in_bud_and_middle_point_of_neck |
| 120 | D135_C | 108.24 | Distance_between_nuclear_brightest_point_in_mother_and_mother_center |
| 121 | D105_A | 108.21 | Ratio_of_D102_to_C103 |
| 122 | D117_A | 107.86 | Distance_between_nuclear_gravity_center_and_cell_center |
| 123 | D14-1_C | 107.76 | Nuclear_size_in_mother |
| 124 | D14-1_A | 107.72 | Nuclear_size |
| 125 | CCV104_A1B | 107.29 | Coefficient_of_variation_of_C104_A1B |
| 126 | CCV114_A1B | 107.28 | Coefficient_of_variation_of_C114_A1B |
| 127 | A7-2_A1B | 107.28 | Size_of_actin_region_in_bud |
| 128 | D207 | 106.72 | nuclear_A1_ratio_to_budded_cells |
| 129 | CCV115_C | 106.32 | Coefficient_of_variation_of_C115_C |
| 130 | CCV110_A1B | 105.90 | Coefficient_of_variation_of_C110_A1B |
| 131 | CCV116_A1B | 105.73 | Coefficient_of_variation_of_C116_A1B |
| 132 | D174_C | 105.69 | Maximal_distance_between_nuclear_gravity_center_and_nuclear_outline_in_bud |
| 133 | CCV111_C | 105.67 | Coefficient_of_variation_of_C111_C |
| 134 | D143_C | 105.14 | Distance_between_nuclear_outline_point_D6-1_in_mother_and_middle_point_of_neck |
| 135 | D179_A | 104.45 | Nuclear_minimum_radius |
| 136 | A7-2_C | 104.22 | Size_of_actin_region_in_bud |
| 137 | D177_C | 104.06 | Nuclear_long_axis_length_in_bud |
| 138 | D114_A1B | 103.76 | Ratio_of_D110_to_C128 |
| 139 | D137_C | 103.17 | Distance_between_nuclear_brightest_point_in_bud_and_bud_center |
| 140 | CCV103_A | 103.13 | Coefficient_of_variation_of_C103_A |
| 141 | D179_C | 103.12 | Nuclear_minimum_radius_in_mother |
| 142 | A101_A1B | 102.68 | Actin_region_ratio_in_whole_cell |
| 143 | D148_C | 102.49 | Relative_distance_of_nuclear_brightest_point_in_mother_to_mother_center |
| 144 | D175_A1B | 102.21 | Maximal_distance_between_nuclear_gravity_center_and_nuclear_outline |
| 145 | D211 | 102.20 | nuclear_A1_ratio_to_nuclear_AA1BC_cells |
| 146 | D109_C | 101.27 | Distance_between_nuclear_gravity_center_in_bud_and_middle_point_of_neck |
| 147 | D169_C | 100.87 | Angle_between_C4-1D1-1_and_C4-1C1 |
| 148 | D178_A1B | 100.79 | Nuclear_long_axis_length |
| 149 | D188_A | 100.74 | Distance_between_nuclear_gravity_center_and_brightest_point |
| 150 | C123_A1B | 100.60 | Small_bud_ratio |
| 151 | CCV126_A1B | 100.59 | Coefficient_of_variation_of_C126_A1B |
| 152 | D121_C | 100.51 | Distance_between_nuclear_gravity_center_in_bud_and_bud_tip |
| 153 | D112_C | 100.42 | Ratio_of_D108_to_C128 |
| 154 | D139_C | 100.40 | Distance_between_nuclear_brightest_point_in_bud_and_bud_tip |
| 155 | D152_A1B | 99.95 | Mobility_of_nucleus_in_mother |
| 156 | D14-2_C | 99.65 | Nuclear_size_in_bud |
| 157 | A112 | 99.63 | actin_bcd_ratio |
| 158 | ACV123_A | 99.57 | Coefficient_of_variation_of_A123_A |
| 159 | CCV106_A1B | 99.38 | Coefficient_of_variation_of_C106_A1B |
| 160 | A8-1_A1B | 99.32 | Total_brightness_of_actin_region_in_mother |
| 161 | CCV107_A1B | 99.29 | Coefficient_of_variation_of_C107_A1B |
| 162 | D119_C | 98.99 | Distance_between_nuclear_gravity_center_in_bud_and_bud_center |
| 163 | CCV104_C | 98.91 | Coefficient_of_variation_of_C104_C |
| 164 | A109_C | 98.55 | Actin_e_ratio |
| 165 | ACV9_C | 98.50 | Coefficient_of_variation_of_A9_C |
| 166 | D176_C | 98.48 | Nuclear_long_axis_length_in_mother |
| 167 | A102_A1B | 98.43 | Bud_actin_region_ratio_to_total_region |
| 168 | D176_A | 98.14 | Nuclear_long_axis_length |
| 169 | ACV104_A1B | 97.96 | Coefficient_of_variation_of_A104_A1B |
| 170 | D144_C | 97.84 | Distance_between_nuclear_outline_point_D6-2_in_bud_and_middle_point_of_neck |
| 171 | D159_C | 97.44 | Angle_between_D2-1D2-2_and_C1-1C1-2 |
| 172 | D106_C | 97.35 | Ratio_of_D103_to_C103 |
| 173 | D158_C | 97.34 | Angle_between_D1-1D1-2_and_C1-1C1-2 |
| 174 | CCV11-1_A1B | 97.30 | Coefficient_of_variation_of_C11-1_A1B |
| 175 | D147_A | 97.14 | Relative_distance_of_nuclear_gravity_center_to_cell_center |
| 176 | C120 | 96.94 | small_bud_ratio |
| 177 | D173_C | 96.67 | Maximal_distance_between_nuclear_gravity_center_and_nuclear_outline_in_mother |
| 178 | A104_A1B | 96.31 | Relative_distance_of_actin_patch_center_from_neck_in_bud |
| 179 | D15-3_A1B | 96.15 | Nuclear_brightness |
| 180 | A117 | 96.12 | actin_d_iso_ratio_to_budded_cells |
| 181 | DCV196_A1B | 95.60 | Coefficient_of_variation_of_D196_A1B |
| 182 | CCV105_A1B | 95.59 | Coefficient_of_variation_of_C105_A1B |
| 183 | D173_A | 95.54 | Maximal_distance_between_nuclear_gravity_center_and_nuclear_outline |
| 184 | C125_A1B | 95.47 | Large_bud_ratio |
| 185 | CCV128_A1B | 95.30 | Coefficient_of_variation_of_C128_A1B |
| 186 | A108_A1B | 95.30 | Actin_d_iso_ratio |
| 187 | D152_C | 94.88 | Mobility_of_nucleus_in_mother |
| 188 | CCV126_A | 94.01 | Coefficient_of_variation_of_C126_A |
| 189 | D15-2_C | 93.97 | Nuclear_brightness_in_bud |
| 190 | D17-3_A1B | 93.89 | Nuclear_fitness_for_ellipse |
| 191 | D15-3_C | 93.79 | Nuclear_brightness_in_whole_cell |
| 192 | D146_C | 93.77 | Distance_between_nuclear_outline_point_D8_in_bud_and_bud_tip |
| 193 | C122 | 93.71 | large_bud_ratio |
| 194 | CCV11-2_A1B | 93.68 | Coefficient_of_variation_of_C11-2_A1B |
| 195 | C11-2_A1B | 93.67 | Bud_cell_size |
| 196 | A120_A1B | 93.65 | Total_length_of_actin_patch_link |
| 197 | C116_C | 93.63 | Axis_ratio_ratio |
| 198 | CCV12-2_A1B | 93.57 | Coefficient_of_variation_of_C12-2_A1B |
| 199 | D17-1_C | 93.53 | Nuclear_fitness_for_ellipse_in_mother |
| 200 | A102_C | 93.36 | Bud_actin_region_ratio_to_total_region |
| 201 | CCV118_A1B | 93.31 | Coefficient_of_variation_of_C118_A1B |
| 202 | DCV197_C | 92.88 | Coefficient_of_variation_of_D197_C |
| 203 | ACV121_A | 92.62 | Coefficient_of_variation_of_A121_A |
| 204 | A116 | 92.38 | actin_c_api_ratio_to_budded_cells |
| 205 | DCV132_A1B | 92.37 | Coefficient_of_variation_of_D132_A1B |
| 206 | A8-2_A1B | 91.89 | Total_brightness_of_actin_region_in_bud |
| 207 | A7-1_A1B | 91.86 | Size_of_actin_region_in_mother |
| 208 | D17-2_C | 91.80 | Nuclear_fitness_for_ellipse_in_bud |
| 209 | D209 | 91.69 | nuclear_C_ratio_to_budded_cells |
| 210 | CCV117_A1B | 91.55 | Coefficient_of_variation_of_C117_A1B |
| 211 | A108_C | 91.46 | Actin_d_iso_ratio |
| 212 | C12-2_A1B | 91.32 | Bud_cell_outline_length |
| 213 | ACV120_A | 91.16 | Coefficient_of_variation_of_A120_A |
| 214 | DCV14-2_C | 91.13 | Coefficient_of_variation_of_D14-2_C |
| 215 | C107_A1B | 90.81 | Long_axis_length_in_bud |
| 216 | ACV7-2_C | 90.81 | Coefficient_of_variation_of_A7-2_C |
| 217 | DCV174_C | 90.76 | Coefficient_of_variation_of_D174_C |
| 218 | D117_C | 90.66 | Distance_between_nuclear_gravity_center_in_mother_and_mother_center |
| 219 | DCV177_C | 90.64 | Coefficient_of_variation_of_D177_C |
| 220 | D17-1_A | 90.45 | Nuclear_fitness_for_ellipse |
| 221 | D172_A1B | 90.15 | Angle_between_C4-1D4_and_C4-1C1 |
| 222 | A8-1_A | 90.09 | Actin_region_brightness |
| 223 | DCV107_A1B | 90.05 | Coefficient_of_variation_of_D107_A1B |
| 224 | DCV139_C | 89.97 | Coefficient_of_variation_of_D139_C |
| 225 | A107 | 89.93 | actin_c_api_ratio |
| 226 | CCV13_A | 89.64 | Coefficient_of_variation_of_C13_A |
| 227 | D170_C | 89.49 | Angle_between_C4-1D2-1_and_C4-1C1 |
| 228 | D162_C | 89.48 | Angle_between_D1-1D1-2_and_C1C4-1 |
| 229 | D188_C | 89.41 | Distance_between_nuclear_gravity_center_and_brightest_point_in_mother |
| 230 | D150_C | 89.18 | Relative_distance_of_nuclear_brightest_point_in_bud_to_bud_center |
| 231 | A112_C | 89.14 | Actin_cd_ratio |
| 232 | A110 | 89.05 | actin_f_ratio |
| 233 | A119 | 88.89 | actin_f_ratio_to_budded_cells |
| 234 | ACV120_A1B | 88.65 | Coefficient_of_variation_of_A120_A1B |
| 235 | DCV143_C | 88.64 | Coefficient_of_variation_of_D143_C |
| 236 | D183_C | 87.59 | Nuclear_axis_ratio_in_bud |
| 237 | A121_A1B | 87.27 | Maximal_distance_between_patches |
| 238 | A110_C | 87.24 | Actin_f_ratio |
| 239 | DCV105_A | 86.71 | Coefficient_of_variation_of_D105_A |
| 240 | D153_C | 86.68 | Mobility_of_nucleus_in_bud |
| 241 | D182_A | 86.66 | Nuclear_axis_ratio |
| 242 | DCV180_C | 86.56 | Coefficient_of_variation_of_D180_C |
| 243 | C106_C | 86.47 | Bud_direction |
| 244 | D123_C | 86.43 | Ratio_of_D121_to_C107 |
| 245 | A121_A | 86.38 | Maximal_distance_between_patches |
| 246 | D15-1_A | 86.25 | Nuclear_brightness |
| 247 | DCV118_A1B | 85.98 | Coefficient_of_variation_of_D118_A1B |
| 248 | A120_A | 85.82 | Total_length_of_actin_patch_link |
| 249 | D165_A1B | 85.76 | Angle_between_D3-1D4-1_and_C1C4-1_or_between_D3-3D4-3_and_C1C4-1 |
| 250 | ACV7-2_A1B | 85.55 | Coefficient_of_variation_of_A7-2_A1B |
| 251 | ACV8-1_A | 85.46 | Coefficient_of_variation_of_A8-1_A |
| 252 | D113_C | 85.27 | Ratio_of_D109_to_C107 |
| 253 | DCV108_C | 84.90 | Coefficient_of_variation_of_D108_C |
| 254 | CCV13_A1B | 84.41 | Coefficient_of_variation_of_C13_A1B |
| 255 | DCV147_A1B | 84.20 | Coefficient_of_variation_of_D147_A1B |
| 256 | DCV198_C | 84.17 | Coefficient_of_variation_of_D198_C |
| 257 | ACV102_A1B | 83.99 | Coefficient_of_variation_of_A102_A1B |
| 258 | ACV121_A1B | 83.82 | Coefficient_of_variation_of_A121_A1B |
| 259 | ACV101_A1B | 83.77 | Coefficient_of_variation_of_A101_A1B |
| 260 | A120_C | 83.58 | Total_length_of_actin_patch_link |
| 261 | D180_C | 83.33 | Nuclear_minimum_radius_in_bud |
| 262 | ACV121_C | 83.02 | Coefficient_of_variation_of_A121_C |
| 263 | A104_C | 82.97 | Relative_distance_of_actin_patch_center_from_neck_in_bud |
| 264 | DCV126_A1B | 82.91 | Coefficient_of_variation_of_D126_A1B |
| 265 | A101_C | 82.49 | Actin_region_ratio_in_whole_cell |
| 266 | A123_A1B | 82.36 | Ratio_of_actin_patches_to_actin_region |
| 267 | DCV151_C | 82.29 | Coefficient_of_variation_of_D151_C |
| 268 | DCV112_C | 82.17 | Coefficient_of_variation_of_D112_C |
| 269 | A7-1_C | 81.78 | Size_of_actin_region_in_mother |
| 270 | DCV104_A1B | 81.72 | Coefficient_of_variation_of_D104_A1B |
| 271 | ACV8-2_A1B | 81.47 | Coefficient_of_variation_of_A8-2_A1B |
| 272 | ACV101_A | 81.04 | Coefficient_of_variation_of_A101_A |
| 273 | DCV17-1_C | 81.02 | Coefficient_of_variation_of_D17-1_C |
| 274 | DCV14-1_C | 80.67 | Coefficient_of_variation_of_D14-1_C |
| 275 | DCV130_C | 80.62 | Coefficient_of_variation_of_D130_C |
| 276 | CCV12-1_A1B | 80.50 | Coefficient_of_variation_of_C12-1_A1B |
| 277 | CCV11-1_C | 80.46 | Coefficient_of_variation_of_C11-1_C |
| 278 | D15-1_C | 80.03 | Nuclear_brightness_in_mother |
| 279 | ACV102_C | 79.95 | Coefficient_of_variation_of_A102_C |
| 280 | D147_C | 79.51 | Relative_distance_of_nuclear_gravity_center_in_mother_to_mother_center |
| 281 | D149_C | 79.49 | Relative_distance_of_nuclear_gravity_center_in_bud_to_bud_center |
| 282 | D151_C | 79.47 | Ratio_of_distance_between_each_nucleus_and_middle_point_of_neck |
| 283 | C110_C | 79.28 | Distance_between_bud_tip_and_mother_long_axis_extension |
| 284 | DCV15-2_C | 78.98 | Coefficient_of_variation_of_D15-2_C |
| 285 | CCV112_A1B | 78.56 | Coefficient_of_variation_of_C112_A1B |
| 286 | D163_C | 78.52 | Angle_between_D2-1D2-2_and_C1C4-1 |
| 287 | ACV7-1_A | 78.40 | Coefficient_of_variation_of_A7-1_A |
| 288 | DCV127_A | 78.29 | Coefficient_of_variation_of_D127_A |
| 289 | A123_A | 78.27 | Ratio_of_actin_patches_to_actin_region |
| 290 | C125 | 77.95 | large_bud_ratio_to_buded_cells |
| 291 | DCV114_A1B | 77.64 | Coefficient_of_variation_of_D114_A1B |
| 292 | A121_C | 77.53 | Maximal_distance_between_patches |
| 293 | DCV146_C | 77.29 | Coefficient_of_variation_of_D146_C |
| 294 | A8-1_C | 77.21 | Total_brightness_of_actin_region_in_mother |
| 295 | DCV179_C | 77.01 | Coefficient_of_variation_of_D179_C |
| 296 | D202 | 76.59 | nuclear_C_ratio |
| 297 | A122_A | 76.48 | Number_of_bright_actin_patches |
| 298 | DCV148_A1B | 76.14 | Coefficient_of_variation_of_D148_A1B |
| 299 | D16-2_C | 76.10 | Maximal_intensity_of_nuclear_brightness_in_bud |
| 300 | CCV101_A1B | 76.05 | Coefficient_of_variation_of_C101_A1B |
| 301 | D197_C | 76.01 | Ratio_of_nuclear_size |
| 302 | C123 | 75.93 | small_bud_ratio_to_budded_cells |
| 303 | ACV123_A1B | 75.90 | Coefficient_of_variation_of_A123_A1B |
| 304 | ACV120_C | 75.89 | Coefficient_of_variation_of_A120_C |
| 305 | DCV181_A1B | 75.76 | Coefficient_of_variation_of_D181_A1B |
| 306 | A118 | 75.67 | actin_e_ratio_to_budded_cells |
| 307 | A107_C | 75.26 | Actin_c_api_ratio |
| 308 | DCV102_A | 75.23 | Coefficient_of_variation_of_D102_A |
| 309 | D166_C | 75.08 | Angle_between_D1-1D1-2_and_C4-1C4-2 |
| 310 | ACV104_C | 75.06 | Coefficient_of_variation_of_A104_C |
| 311 | D198_C | 74.98 | Ratio_of_nuclear_brightness |
| 312 | A122_C | 74.91 | Number_of_bright_actin_patches |
| 313 | DCV141_C | 74.46 | Coefficient_of_variation_of_D141_C |
| 314 | DCV113_C | 74.33 | Coefficient_of_variation_of_D113_C |
| 315 | CCV113_C | 74.09 | Coefficient_of_variation_of_C113_C |
| 316 | DCV106_C | 74.01 | Coefficient_of_variation_of_D106_C |
| 317 | DCV182_A | 73.77 | Coefficient_of_variation_of_D182_A |
| 318 | CCV109_A1B | 73.76 | Coefficient_of_variation_of_C109_A1B |
| 319 | A122_A1B | 73.73 | Number_of_bright_actin_patches |
| 320 | CCV110_C | 73.53 | Coefficient_of_variation_of_C110_C |
| 321 | DCV17-1_A | 73.51 | Coefficient_of_variation_of_D17-1_A |
| 322 | CCV105_C | 73.45 | Coefficient_of_variation_of_C105_C |
| 323 | DCV152_A1B | 73.41 | Coefficient_of_variation_of_D152_A1B |
| 324 | DCV131_C | 73.19 | Coefficient_of_variation_of_D131_C |
| 325 | DCV194_A | 73.05 | Coefficient_of_variation_of_D194_A |
| 326 | DCV145_C | 72.91 | Coefficient_of_variation_of_D145_C |
| 327 | DCV110_A1B | 72.76 | Coefficient_of_variation_of_D110_A1B |
| 328 | A109 | 72.68 | actin_e_ratio |
| 329 | CCV126_C | 72.44 | Coefficient_of_variation_of_C126_C |
| 330 | CCV113_A1B | 72.14 | Coefficient_of_variation_of_C113_A1B |
| 331 | DCV109_C | 72.09 | Coefficient_of_variation_of_D109_C |
| 332 | DCV14-3_A1B | 72.01 | Coefficient_of_variation_of_D14-3_A1B |
| 333 | DCV179_A | 71.94 | Coefficient_of_variation_of_D179_A |
| 334 | D161_A1B | 71.80 | Angle_between_D3-1D4-1_and_C1-1C1-2_or_between_D3-3D4-3_and_C1-1C1-2 |
| 335 | D189_C | 71.66 | Distance_between_nuclear_gravity_center_and_brightest_point_in_bud |
| 336 | DCV116_C | 71.47 | Coefficient_of_variation_of_D116_C |
| 337 | A106 | 71.46 | actin_b_ratio |
| 338 | D192_C | 71.23 | Average_of_nuclear_brightness_in_bud |
| 339 | DCV195_C | 71.23 | Coefficient_of_variation_of_D195_C |
| 340 | DCV121_C | 71.14 | Coefficient_of_variation_of_D121_C |
| 341 | C113_A1B | 70.87 | Distance_between_bud_tip_and_mother_long_axis_through_middle_point_of_neck |
| 342 | DCV145_A1B | 70.84 | Coefficient_of_variation_of_D145_A1B |
| 343 | CCV103_A1B | 70.73 | Coefficient_of_variation_of_C103_A1B |
| 344 | D196_A1B | 70.56 | Maximal_intensity_of_nuclear_brightness_divided_by_average |
| 345 | DCV152_C | 70.17 | Coefficient_of_variation_of_D152_C |
| 346 | D16-3_C | 69.97 | Maximal_intensity_of_nuclear_brightness_in_whole_cell |
| 347 | DCV128_C | 69.96 | Coefficient_of_variation_of_D128_C |
| 348 | D190_A1B | 69.85 | Distance_between_nuclear_gravity_center_and_brightest_point |
| 349 | CCV107_C | 69.81 | Coefficient_of_variation_of_C107_C |
| 350 | DCV103_C | 69.77 | Coefficient_of_variation_of_D103_C |
| 351 | D201 | 69.59 | nuclear_B_ratio |
| 352 | D191_C | 69.17 | Average_of_nuclear_brightness_in_mother |
| 353 | DCV144_C | 69.13 | Coefficient_of_variation_of_D144_C |
| 354 | DCV134_C | 69.00 | Coefficient_of_variation_of_D134_C |
| 355 | D193_C | 68.62 | Average_of_nuclear_brightness_in_whole_cell |
| 356 | D208 | 68.48 | nuclear_B_ratio_to_budded_cells |
| 357 | ACV8-2_C | 68.40 | Coefficient_of_variation_of_A8-2_C |
| 358 | CCV108_C | 68.34 | Coefficient_of_variation_of_C108_C |
| 359 | CCV12-1_C | 68.28 | Coefficient_of_variation_of_C12-1_C |
| 360 | ACV101_C | 68.25 | Coefficient_of_variation_of_A101_C |
| 361 | D167_C | 67.58 | Angle_between_D2-1D2-2_and_C4-1C4-2 |
| 362 | D16-1_C | 67.56 | Maximal_intensity_of_nuclear_brightness_in_mother |
| 363 | CCV11-2_C | 67.43 | Coefficient_of_variation_of_C11-2_C |
| 364 | CCV102_A1B | 67.25 | Coefficient_of_variation_of_C102_A1B |
| 365 | DCV173_A | 67.25 | Coefficient_of_variation_of_D173_A |
| 366 | DCV123_C | 67.15 | Coefficient_of_variation_of_D123_C |
| 367 | C121 | 67.11 | medium_bud_ratio |
| 368 | DCV170_A1B | 67.02 | Coefficient_of_variation_of_D170_A1B |
| 369 | D154_A | 66.92 | Angle_between_C1D1-1_and_C1C1-2 |
| 370 | C127_A | 66.71 | Thickness_difference_of_cell_wall |
| 371 | CCV108_A1B | 66.63 | Coefficient_of_variation_of_C108_A1B |
| 372 | D214 | 66.45 | nuclear_A1_ratio_to_nuclear_A1BC_cells |
| 373 | A103_A1B | 66.41 | Relative_distance_of_actin_patch_center_from_neck_in_mother |
| 374 | C125_C | 66.14 | Large_bud_ratio |
| 375 | DCV117_A | 66.10 | Coefficient_of_variation_of_D117_A |
| 376 | DCV176_C | 66.03 | Coefficient_of_variation_of_D176_C |
| 377 | C124_C | 66.02 | Medium_bud_ratio |
| 378 | CCV118_C | 66.00 | Coefficient_of_variation_of_C118_C |
| 379 | DCV172_A1B | 65.99 | Coefficient_of_variation_of_D172_A1B |
| 380 | D182_C | 65.68 | Nuclear_axis_ratio_in_mother |
| 381 | CCV117_C | 65.27 | Coefficient_of_variation_of_C117_C |
| 382 | DCV188_A | 65.01 | Coefficient_of_variation_of_D188_A |
| 383 | DCV14-3_C | 64.84 | Coefficient_of_variation_of_D14-3_C |
| 384 | DCV165_A1B | 64.46 | Coefficient_of_variation_of_D165_A1B |
| 385 | DCV161_A1B | 63.98 | Coefficient_of_variation_of_D161_A1B |
| 386 | A110_A1B | 63.86 | Actin_f_ratio |
| 387 | ACV8-1_C | 63.71 | Coefficient_of_variation_of_A8-1_C |
| 388 | DCV14-1_A | 63.70 | Coefficient_of_variation_of_D14-1_A |
| 389 | ACV7-1_C | 63.61 | Coefficient_of_variation_of_A7-1_C |
| 390 | A113_C | 63.60 | Actin_n_ratio |
| 391 | DCV173_C | 63.60 | Coefficient_of_variation_of_D173_C |
| 392 | D154_C | 63.58 | Angle_between_C1D1-1_and_C1C1-2 |
| 393 | C124 | 63.32 | medium_bud_ratio_to_buded_cells |
| 394 | DCV135_A | 63.32 | Coefficient_of_variation_of_D135_A |
| 395 | CCV128_C | 63.21 | Coefficient_of_variation_of_C128_C |
| 396 | DCV176_A | 63.18 | Coefficient_of_variation_of_D176_A |
| 397 | D216 | 63.06 | nuclear_C_ratio_to_nuclear_A1BC_cells |
| 398 | CCV112_C | 63.05 | Coefficient_of_variation_of_C112_C |
| 399 | DCV158_C | 62.73 | Coefficient_of_variation_of_D158_C |
| 400 | DCV159_C | 62.68 | Coefficient_of_variation_of_D159_C |
| 401 | CCV106_C | 62.44 | Coefficient_of_variation_of_C106_C |
| 402 | D16-1_A | 62.41 | Maximal_intensity_of_nuclear_brightness |
| 403 | DCV125_C | 62.38 | Coefficient_of_variation_of_D125_C |
| 404 | A103_C | 62.19 | Relative_distance_of_actin_patch_center_from_neck_in_mother |
| 405 | DCV169_A1B | 62.06 | Coefficient_of_variation_of_D169_A1B |
| 406 | DCV143_A1B | 62.02 | Coefficient_of_variation_of_D143_A1B |
| 407 | A112_A1B | 61.95 | Actin_cd_ratio |
| 408 | A113_A | 61.89 | Actin_n_ratio |
| 409 | DCV185_C | 61.74 | Coefficient_of_variation_of_D185_C |
| 410 | A113_A1B | 61.47 | Actin_n_ratio |
| 411 | DCV147_A | 61.40 | Coefficient_of_variation_of_D147_A |
| 412 | DCV17-3_A1B | 61.24 | Coefficient_of_variation_of_D17-3_A1B |
| 413 | A109_A1B | 60.97 | Actin_e_ratio |
| 414 | C124_A1B | 60.89 | Medium_bud_ratio |
| 415 | ACV122_A1B | 60.78 | Coefficient_of_variation_of_A122_A1B |
| 416 | A123_C | 60.49 | Ratio_of_actin_patches_to_actin_region |
| 417 | CCV13_C | 60.39 | Coefficient_of_variation_of_C13_C |
| 418 | DCV154_C | 60.18 | Coefficient_of_variation_of_D154_C |
| 419 | DCV16-1_A | 60.09 | Coefficient_of_variation_of_D16-1_A |
| 420 | DCV154_A | 59.99 | Coefficient_of_variation_of_D154_A |
| 421 | D191_A | 59.98 | Average_of_nuclear_brightness |
| 422 | ACV123_C | 59.59 | Coefficient_of_variation_of_A123_C |
| 423 | DCV15-3_A1B | 59.33 | Coefficient_of_variation_of_D15-3_A1B |
| 424 | ACV122_A | 59.28 | Coefficient_of_variation_of_A122_A |
| 425 | DCV16-3_A1B | 59.22 | Coefficient_of_variation_of_D16-3_A1B |
| 426 | DCV153_C | 59.21 | Coefficient_of_variation_of_D153_C |
| 427 | D193_A1B | 59.05 | Average_of_nuclear_brightness |
| 428 | DCV193_A1B | 58.90 | Coefficient_of_variation_of_D193_A1B |
| 429 | D16-3_A1B | 58.11 | Maximal_intensity_of_nuclear_brightness |
| 430 | D212 | 58.06 | nuclear_B_ratio_to_nuclear_AA1BC_cells |
| 431 | DCV148_A | 57.79 | Coefficient_of_variation_of_D148_A |
| 432 | DCV15-1_C | 57.69 | Coefficient_of_variation_of_D15-1_C |
| 433 | CCV12-2_C | 57.69 | Coefficient_of_variation_of_C12-2_C |
| 434 | C127_A1B | 57.57 | Thickness_difference_of_cell_wall |
| 435 | DCV191_A | 57.12 | Coefficient_of_variation_of_D191_A |
| 436 | CCV109_C | 57.10 | Coefficient_of_variation_of_C109_C |
| 437 | CCV101_C | 56.61 | Coefficient_of_variation_of_C101_C |
| 438 | DCV184_A1B | 56.60 | Coefficient_of_variation_of_D184_A1B |
| 439 | DCV183_C | 56.42 | Coefficient_of_variation_of_D183_C |
| 440 | ACV103_C | 56.15 | Coefficient_of_variation_of_A103_C |
