# Supplementary figures and images for: Single-cell phenomics reveals intra-species variation of phenotypic noise in yeast
Source: BMC Syst Biol. 2013 Jul 3;7:54. doi: 10.1186/1752-0509-7-54 (PMC3711934; doi:10.1186/1752-0509-7-54)

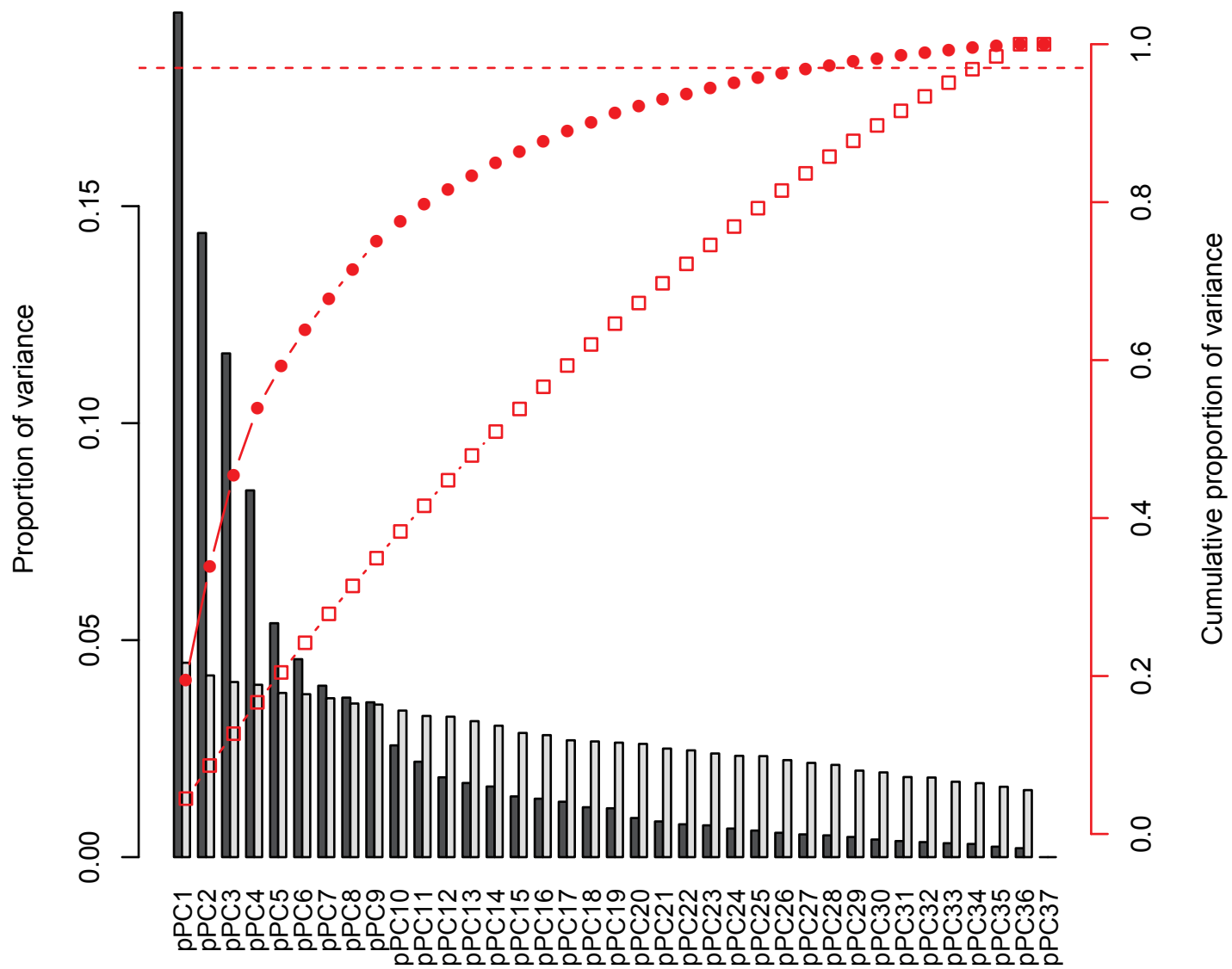

Supplementary Figure 1

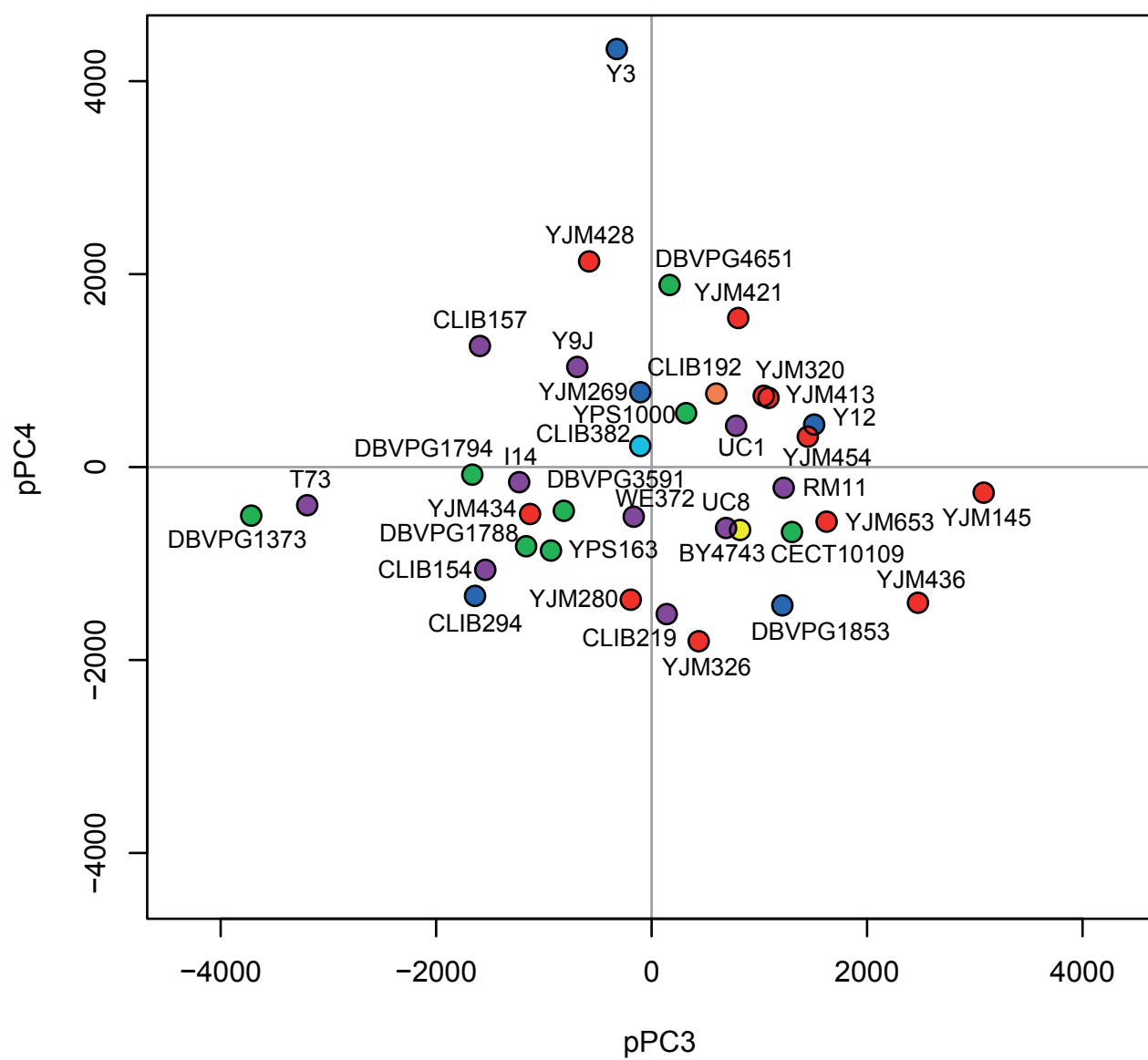

Supplementary Figure 2

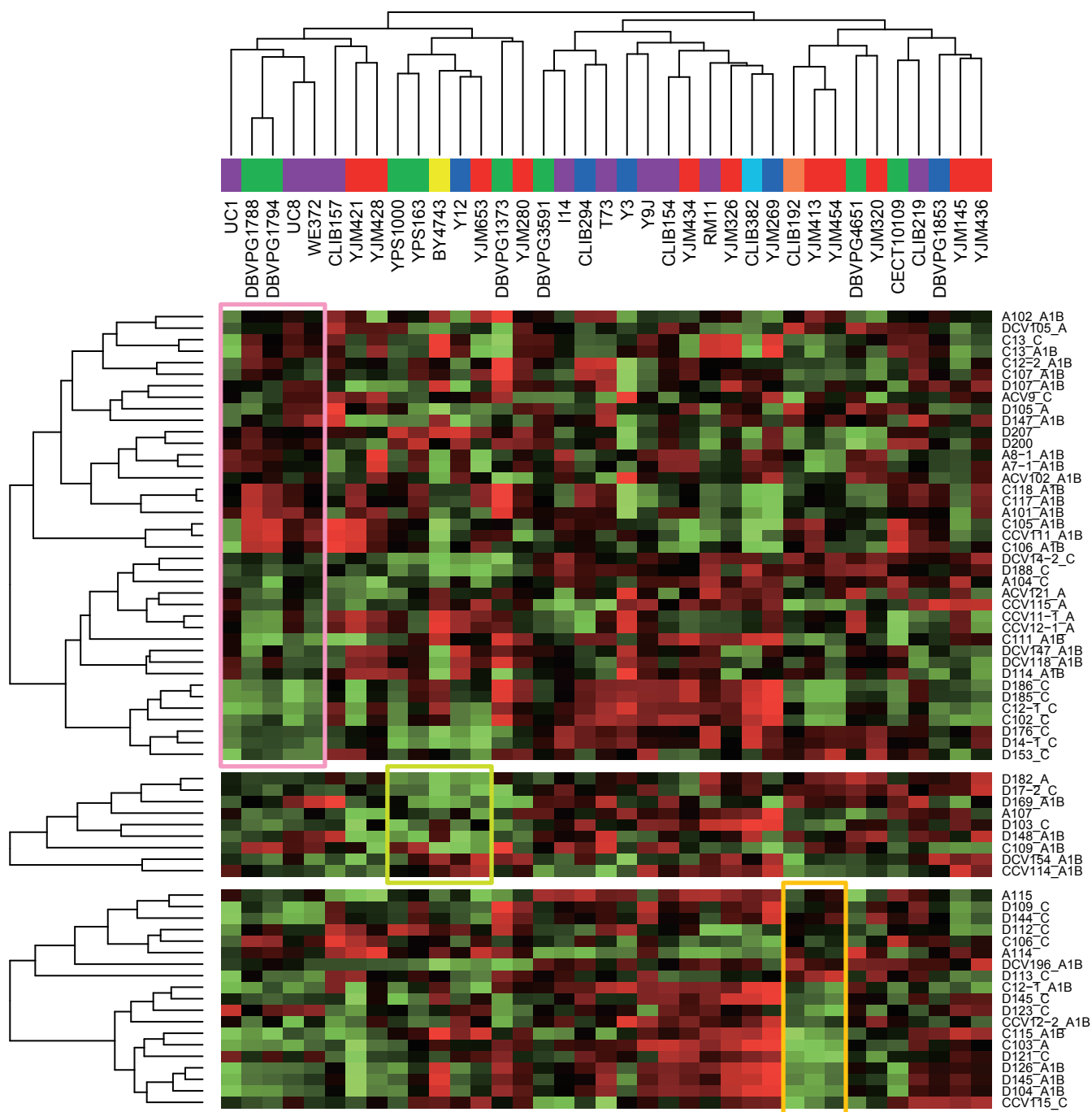

Supplementary Figure 3



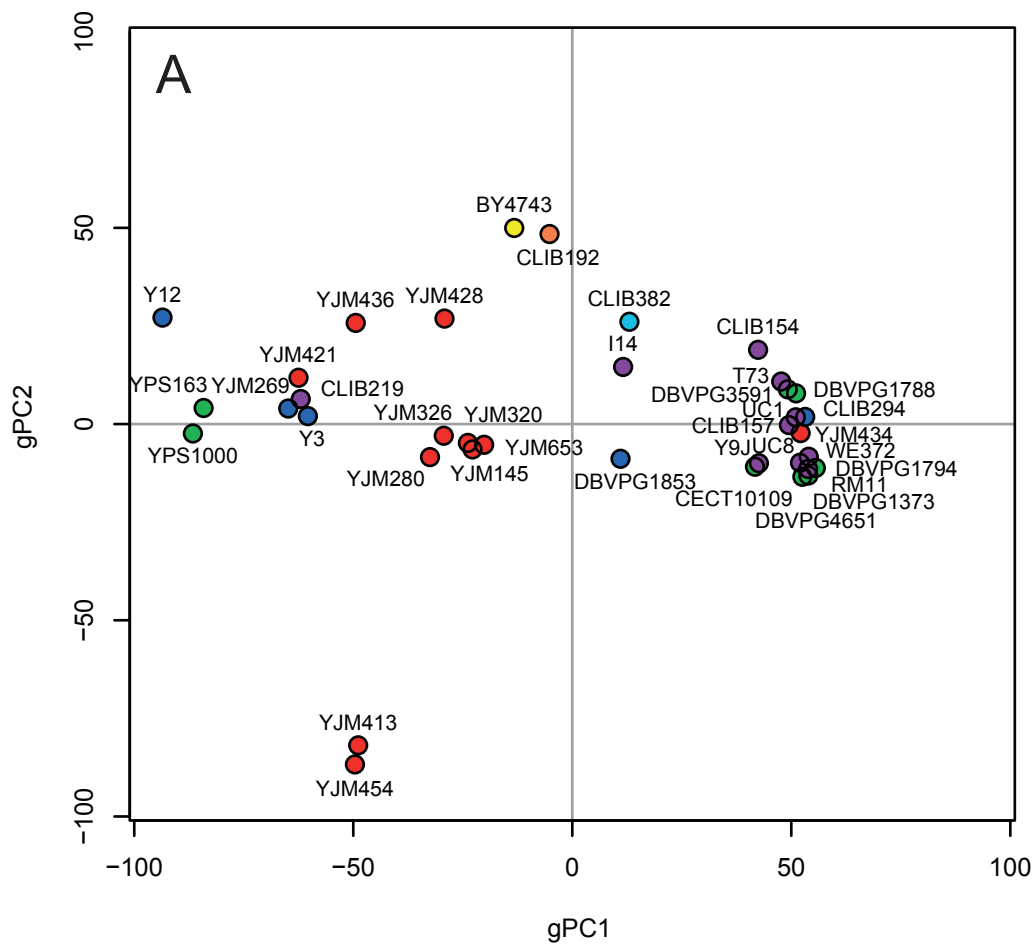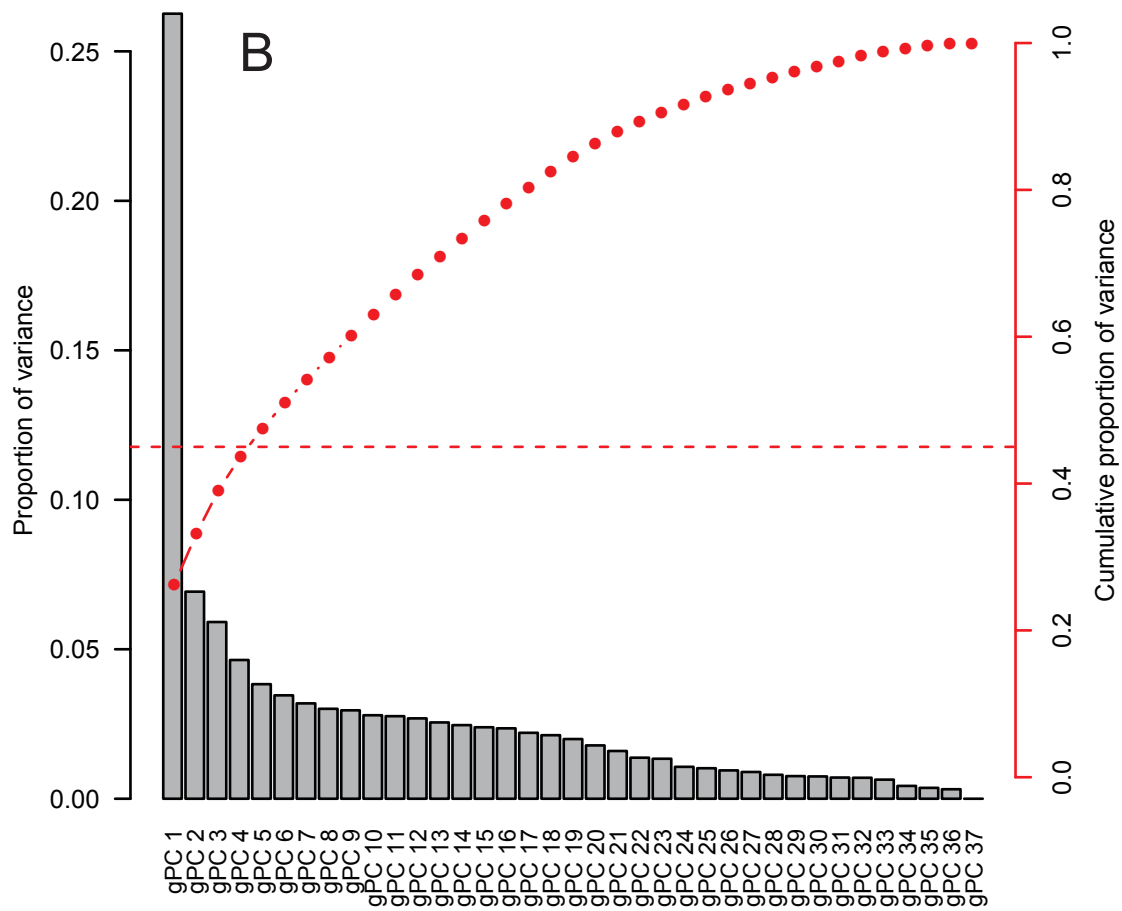

Supplementary Figure 5

Supplement: Additional file 3: Figure S1 — Cumulative proportion of variance of the principal component analysis for the phenotypes. Black and grey bars indicate the proportion of variance (left axis) explained by the pPCs without randomization and after randomization, respectively. Red circles and rectangles indicate the cumulative proportion of variance (right axis) explained by the pPCs without randomization and after randomization, respectively. The horizontal dashed red line indicates 97% of the cumulative proportion of variance. Figure S2. Principal component analysis of S. cerevisiae morphological variation. Dots represent strains by their coordinates along principal components pPC3 and pPC4, from the same PCA analysis as in Figure 2A. Figure S3. Heatmap of the rank-sum values of the parameters contributing to discriminate strain classes I, II and III by LDA. The dendrogram and strain labels at the top are the same as in Figure 3A. Three heatmaps indicate the rank-sum values of the representative parameters for the class I, II and III from top to bottom, respectively. Red, black, and green, indicate high, middle and low values, respectively. Pink, greenyellow and lightorange rectangles on the heatmap indicate the class I, II and III of strains, respectively. Figure S4. Strains distribution along the parameters representing the morphological features of each class. Pink, greenyellow, lightorange and black circles indicate strains of classes I, II, III and others, respectively. Red frames indicate scatter plots of the distribution of the 37 strains on the representative parameters for each class. A) Parameters representative of Class I. B) Parameters representative of Class II. C) Parameters representative of Class III. Figure S5. Principal component analysis of S. cerevisiae genetic variation. A boolean matrix of the single nucleotide polymorphisms (Schacherer et al. [19]) was used for principal component analysis. A) Strains are represented by their coordinates along the first two principal components [file 1752-0509-7-54-S3.pdf]
